# CYLD-mediated lysine63 deubiquitination regulates synaptic transmission and autophagy to mitigate age-related sequelae

**DOI:** 10.1038/s41467-026-73966-5

**Published:** 2026-06-04

**Authors:** Aggeliki Sotiriou, Georgios Konstantinidis, Nektarios Tavernarakis

**Affiliations:** 1 Institute of Molecular Biology and Biotechnology, Foundation of Research and Technology–Hellas, Heraklion, Crete Greece; 2 Department of Basic Sciences, School of Medicine, University of Crete, Heraklion, Crete Greece

**Keywords:** Ageing, Macroautophagy, Ubiquitylation, Cellular neuroscience

## Abstract

Lysine63 polyubiquitination is a prevalent post-translational modification in the central nervous system. Deficiency of CYLD, a lysine63-specific deubiquitinase, is linked to synaptic dysfunction and neurodegenerative disorders. However, our understanding of how CYLD contributes to the manifestation of neuronal deficits, particularly in the context of ageing, remains limited. Here, we report that CYLD-1 is essential for physiological lifespan in the nematode *Caenorhabditis elegans*. Neuronal CYLD-1 supports cholinergic neurotransmission and GABAergic synapse integrity, ensuring intact locomotory capacity, as well as learning and memory competence. Specifically, the deubiquitinase activity of CYLD-1 is necessary for upholding cholinergic neurotransmission and lifespan. We further show that CYLD-1 regulates autolysosomal and lysosomal network organisation in neurons and peripheral tissues in vivo. Our work unveils a crucial role of CYLD-1 in optimizing neural activity and behavioural outcomes, to improve organismal fitness and survival.

## Abstract (web summary)

The proteostasis network supports healthy ageing, yet specific mechanisms remain poorly defined. Here the authors show that a conserved lysine deubiquitinase regulates autophagy-lysosomal homeostasis to sustain neural function and lifespan.

## Introduction

Ageing is a biological process characterised by progressive decline in physiological functions and increased susceptibility to age-associated pathologies, including neurodegenerative disorders such as Alzheimer’s disease and other dementias^1^. Neurons, being terminally differentiated, post-mitotic and highly compartmentalised cells, rely heavily on proteostatic mechanisms to preserve cellular integrity^2,3^. Central to maintaining neuronal homoeostasis is the ubiquitin system, a highly conserved molecular machinery that orchestrates protein turnover and signalling cascades^4–6^. The complexity and specificity of ubiquitin signalling is underscored by the diverse types of ubiquitin linkages. Substrate residues, such as lysine, serine, threonine, and the N-terminal methionine, are modified by mono-ubiquitin or polyubiquitin chains^7^. Different types of lysine polyubiquitin chains adopt various conformations, which are recognised by specialised effector proteins that elicit distinct biological outcomes for their substrates. Lysine63 (K63) polyubiquitination, a prominent post-translational modification in neurons, facilitates proteasome-independent proteolysis through the autophagy-lysosome system, as well as non-degradative functions in various signalling pathways, including the DNA damage response, inflammation, and immune responses^8–11^.

The dynamic regulation of ubiquitination is mediated by ubiquitin ligases, which catalyse the transfer of ubiquitin to target proteins, and deubiquitinases (DUBs), which counteract ubiquitination by removing ubiquitin from substrates or cleaving within ubiquitin chains. DUBs comprise over 100 enzymes in the mammalian genome and exhibit remarkable specificity, distinguish between different types of polyubiquitin chain linkages, and operate within various intracellular compartments^12–14^. DUBs in the nervous system are involved in coordinating axon guidance, neuronal survival, and multiple aspects of synaptic biology, including development, function, transmission, and plasticity^15^. These enzymes have also been implicated in the pathogenesis of neurodevelopmental and neurodegenerative disorders^16^.

Cylindromatosis tumour suppressor protein (CYLD) is a DUB that exhibits specificity towards methionine1 (M1) and K63 polyubiquitin chains. Mutations in CYLD were initially identified in patients with familial cylindromatosis, an autosomal dominant disorder that predisposes individuals to multiple tumours known as cylindromas^17,18^. CYLD acts as a negative regulator of the nuclear factor kappa B (NF-κB) transcription factor by deubiquitinating its upstream modulators, the tumour necrosis factor (TNF) receptor-associated factor 2 (TRAF2), TRAF6, and the NF-κB essential modifier (NEMO)/inhibitor of κB (IκB) kinase-γ (IKKγ), thereby impacting physiological processes, including immunity, inflammation, cell proliferation, survival, and death^19^. In addition to its DUB domain, CYLD contains three N-terminal, cytoskeleton-associated protein glycine-rich (CAP-Gly) regions, which facilitate interaction with microtubules. In primary keratinocytes, CYLD interacts with tubulin and the microtubule-associated histone deacetylase 6 (HDAC6), through the CAP-Gly domains, to inhibit the deacetylase activity of HDAC6 and enhance α-tubulin acetylation, thereby promoting microtubule assembly and stability^20^.

Despite its abundant expression in the mammalian brain, the role of CYLD in the nervous system remains largely unexplored^21^. CYLD is enriched in synapses and accumulates in post-synaptic densities upon neuronal activity^22,23^. Recent studies have shown that CYLD deficiency is associated with structural dendritic abnormalities, disrupted synaptic plasticity, and behavioural deficits in mice, ranging from anxiety and autistic-like behaviours to memory impairment^24–28^. Notably, CYLD variants have been identified in patients with neurodegenerative diseases, such as amyotrophic lateral sclerosis and Alzheimer’s disease, highlighting its significance in maintaining nervous system integrity and its potential role in neurodegenerative pathology^29–31^.

In this study, we demonstrate that CYLD deubiquitinase activity is crucial for the preservation of neuronal physiology, including locomotory and cognitive functions, during ageing in *Caenorhabditis elegans*. Furthermore, neuronal CYLD is a key mediator of longevity in the nematode. Mechanistically, CYLD-1 exerts its effects on neuronal function and lifespan by promoting autophagic recycling and lysosomal homoeostasis in the nervous system and other tissues.

## Results

### CYLD-1 deubiquitinase activity promotes longevity

**Fig. 1:**
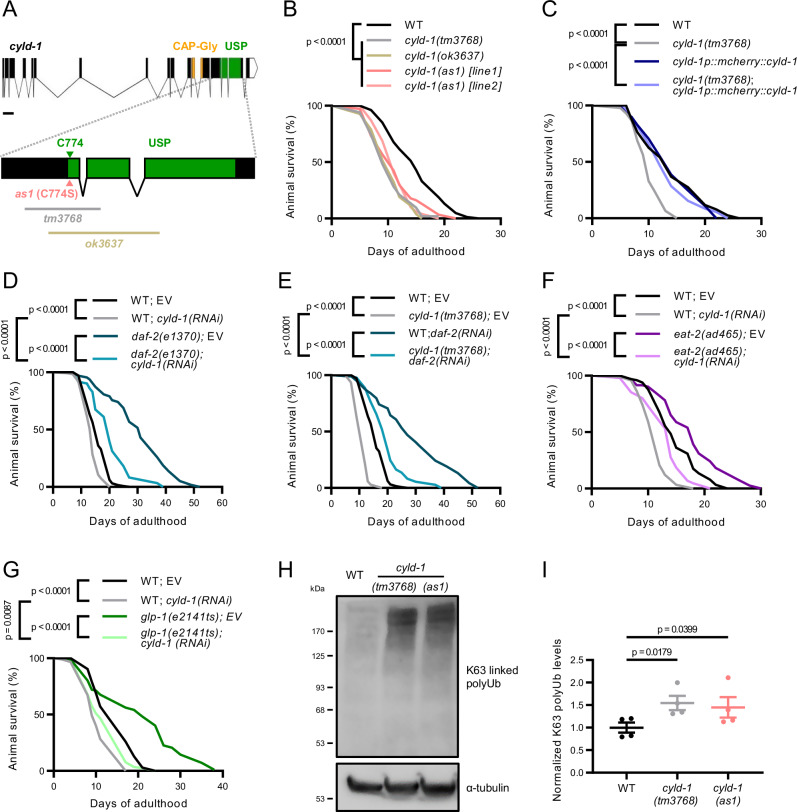
CYLD-1 regulates lifespan through its catalytic activity. **A** Schematic representation of the *cyld-1* genetic locus. Filled boxes represent exons and lines represent splicing events proportional to intron length. Orange boxes mark the cytoskeleton-associated protein glycine-rich (CAP-Gly) domain, and green boxes mark the ubiquitin-specific protease (USP) catalytic domain. The green arrowhead indicates the conserved catalytic cysteine 774 (C774). The grey line denotes the deletion of the *tm3768* variant. The gold line denotes the deletion of the *ok3637* variant. The *as1* cysteine 774 to serine (C774S) variant is denoted with pink colour and arrowhead. Scale bar, 500 bp at the upper panel. The exon-intron graphic element was generated via the Exon-Intron Graphic Maker web app by Nikhil Bhatla, 2012, available at http://wormweb.org/exonintron. **B** Survival curves of WT, *cyld-1(tm3768)*, *cyld-1(ok3637)* and *cyld-1(as1)* worms; log rank, Mantel-Cox test. **C** Survival curves of WT, *cyld-1(tm3768)*, *cyld-1p::mcherry::cyld-1* and *cyld-1(tm3768); cyld-1p::mcherry::cyld-1* worms; log rank, Mantel-Cox test. **D** Survival curves of WT and *daf-2(e1370)* worms, treated with control (empty vector, EV) or *cyld-1(RNAi)* from hatching; log rank, Mantel-Cox test. **E** Survival curves of WT and *cyld-1(tm3768)* worms, treated with control (EV) or *daf-2(RNAi)* from hatching; log rank, Mantel-Cox test. **F** Survival curves of WT and *eat-2(ad465)* worms, treated with control (EV) or *cyld-1(RNAi)* from hatching; log rank, Mantel-Cox test. **G** Survival curves of WT and *glp-1(e2141ts)* worms, treated with control (EV) or *cyld-1(RNAi)* from hatching; log rank, Mantel-Cox test. **H** Immunoblot analysis of lysates from D1 WT, *cyld-1(tm3768)* and *cyld-1(as1)* worms, detecting endogenous K63-linked polyubiquitin (polyUb) chains and α-tubulin. **I** Quantification of the K63-linked polyubiquitin levels normalised to α-tubulin from (**H**) (*N* = 4 experiments; One-way ANOVA with Dunnettt’s multiple comparison test). Data presented as mean ± SEM. Source data are provided as a Source Data file.

To investigate the potential role of CYLD in regulating lifespan and ageing, we used the nematode *C. elegans*, a versatile model for studying the genetic control of ageing^32^. The nematode harbours a single homologue of mammalian CYLD, which alleviates potential redundancy considerations. The *C. elegans cyld-1(tm3768*) mutant carries a 496 bp deletion that includes the conserved C-terminal ubiquitin-specific protease (USP) catalytic domain (Fig. 1A). We observed that *cyld-1(tm3768)* mutant animals have a shorter lifespan compared to wild-type (WT) nematodes (Fig. 1B). In addition, we have characterised and outcrossed a second *cyld-1* variant *(ok3637)* that carries an 858 bp deletion that includes the USP catalytic domain (Fig. 1A). This independent *cyld-1* variant exhibited identically reduced lifespan compared to the *cyld-1(tm3768*) mutant animals (Fig. 1B). Restoring *cyld-1* expression, under its endogenous promoter, rescued the shortened lifespan in *cyld-1(tm3768)* nematodes, confirming the direct relationship between CYLD-1 deficiency and compromised longevity (Fig. 1C). On the other hand, overexpression of CYLD-1 in wild type genetic background was not sufficient to extend lifespan (Fig. 1C).

Genetic perturbations impinging on endocrine signalling are a well-established mechanism for extending lifespan^33^. The *daf-2* gene encodes the insulin and insulin-like growth factor 1 (IGF-1) receptor in *C. elegans*, and *daf-2* deficiency significantly increases animal lifespan^34^. We observed a marked reduction in the lifespan of *daf-2(e1370)* mutants upon *cyld-1* silencing (Fig. 1D). Similarly, *daf-2* knockdown in *cyld-1(tm3768)* mutants failed to prolong lifespan to the same extent as in WT animals (Fig. 1E). Impairment of pharyngeal pumping [*eat-2(ad465)*] leading to reduced rate of food uptake or elimination of the germline stem cells [*glp-1(e2141ts)*] leading to sterility, represent two independent paradigms of lifespan extension in *C. elegans*^35,36^. Depletion of *cyld-1* shortens lifespan in both of these longevity paradigms as well (Fig. 1F, G). These results suggest that CYLD-1 is essential for normal lifespan and for lifespan extension mediated by disruptions in the insulin/IGF-1 signalling, calorie restriction or reproductive curtailment. Notably, CYLD-1 appears to act in parallel or independent pathway(s) from those responsible for the extended lifespan of the longevity paradigms (Fig. 1D–G).

To confirm that CYLD-1 functions as a K63-specific DUB in vivo in *C. elegans*, we compared K63 polyubiquitination levels between WT and *cyld-1(tm3768)* mutant nematodes. Immunoblot analysis using an antibody specific to K63-linked di-ubiquitin revealed elevated K63 polyubiquitination levels in *cyld-1(tm3768)* animals compared to WT (Fig. 1H, I). In addition to its catalytic domain, CYLD-1 contains an N-terminal CAP-Gly domain (Fig. 1A). In mammals, the CAP-Gly domains of CYLD mediate interaction with microtubules and IKKγ^20,37,38^. While *C. elegans* lacks NF-κB nuclear effectors and NF-κB signalling, the regulation of the microtubule cytoskeleton by CYLD-1 could impact animal lifespan. To specifically target CYLD-1 catalytic activity, we used CRISPR mutagenesis to abrogate its DUB function. We substituted the conserved catalytic cysteine 774 with serine, generating a catalytically inactive CYLD-1^C774S^ mutant, designated as *cyld-1(as1)* (Fig. 1A). Two independent lines were generated and outcrossed with WT animals to eliminate potential off-target mutations. Nematodes expressing catalytic-dead CYLD-1^C774S^ exhibited increased K63 polyubiquitination, corroborating the role of CYLD-1 as a K63-specific DUB in vivo (Fig. 1H, I). Notably, *cyld-1(as1)* mutants expressing endogenous, catalytically inactive CYLD-1 showed reduced lifespan, similar to the *cyld-1(tm3768)* deletion mutants (Fig. 1B). These findings indicate that the DUB activity of CYLD-1 is critical for longevity.

### CYLD-1 functions as a K63-specific DUB in the nervous system

**Fig. 2:**
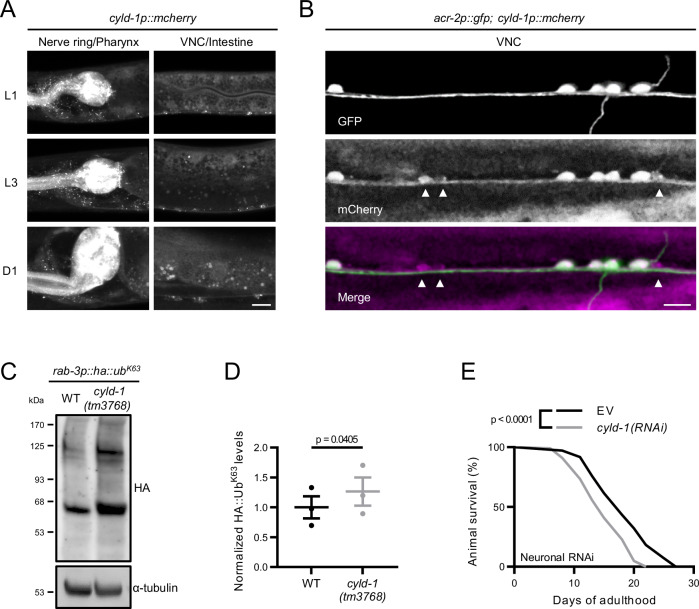
Expression and function of CYLD-1 in the nervous system. **A** Confocal microscopy images (maximum projection) of *cyld-1p::mcherry* worms of the indicated developmental stages. mCherry fluorescence driven by the *cyld-1* promoter in the head and midbody areas reveals nerve ring and VNC expression. Scale bar, 10 μm. **B** Widefield microscopy images of the VNC of D1 *acr-2p::gfp; cyld-1p::mcherry* worms, showing GFP fluorescence in A- and B-type cholinergic motor neurons and mCherry fluorescence driven by the *cyld-1* promoter. Arrowheads indicate mCherry-only (*cyld-1*) expressing motor neurons. Scale bar, 10 μm. **C** Immunoblot analysis of lysates from D1 WT and *cyld-1(tm3768)* worms expressing *ha::ub*^K63^ under the control of the *rab-3* promoter, detecting HA-tagged neuronal K63-linked polyubiquitin chains and α-tubulin. **D** Quantification of the HA-tagged neuronal K63-linked polyubiquitin levels normalised to α-tubulin from (**C**) (*N* = 3 experiments; Two-sided Student’s *t* test). **E** Survival curves of *sid-1(qt9); rgef-1p::gfp; rgef-1p::sid-1* worms (strain MAH677) treated with control (EV) or *cyld-1(RNAi)* from hatching; log rank, Mantel-Cox test. Data presented as mean ± SEM. Source data are provided as a Source Data file.

Accumulating evidence highlights the tissue-specific role of CYLD in regulating K63 polyubiquitination in the mammalian nervous system^24–26^. To determine whether CYLD-1 is expressed in the *C. elegans* nervous system, we generated transgenic animals expressing soluble mCherry under the control of the *cyld-1* promoter (*cyld-1p::mcherry*). During larval development, we observed *cyld-1* expression in neurons of the nerve ring, the ventral nerve cord (VNC), and in the pharynx (Fig. 2A). In adult worms, the *cyld-1* promoter was also active in the intestine (Fig. 2A and Supplementary Fig. S1A). mCherry-positive neuronal cell bodies were located in the VNC (Fig. 2B), the primary nerve cord, which contains the cell bodies of cholinergic and GABAergic motor neurons responsible for controlling movement^39^. To define specific neuronal types expressing *cyld-1* (mCherry) within the VNC, we generated transgenic nematodes co-expressing *cyld-1p::mcherry* and GFP under the control of the cholinergic-specific promoter *acr-2* (*acr-2p::gfp*). In the VNC, *cyld-1* expression was detected in GFP-positive cell bodies corresponding to A- and B-type cholinergic motor neurons (Fig. 2B)^40,41^, as well as in additional GFP-negative neuronal cell bodies in the VNC (Fig. 2B, arrowheads), which may correspond to AS cholinergic or D-type GABAergic motor neurons^42^.

To examine whether CYLD-1 functions as a K63-specific DUB in the *C. elegans* nervous system, we generated transgenic animals expressing HA-tagged ubiquitin exclusively in neurons, under the control of the neuronal-specific promoter *rab-3*. Ubiquitin molecules can be conjugated at any of the seven internal lysines to form polyubiquitin chains of different linkage types. We used a mutant ubiquitin variant (Ub^K63^), in which all lysine residues were substituted with arginine, except for K63 *(rab-3p::ha::ub*^*K63*^) (Supplementary Fig. S1C)^43^. We observed HA::Ub^K63^ incorporation into polyubiquitin chains in neurons of transgenic animals (Supplementary Fig. S1D). Notably, *cyld-1(tm3768)* mutants displayed higher levels of HA::Ub^K63^-modified neuronal proteins, compared to WT animals (Fig. 2C, D), indicating that CYLD-1 regulates K63 ubiquitination in the nervous system.

Given the prominent neuronal expression and DUB activity of CYLD-1, we sought to evaluate its neuronal contribution to animal longevity. Neuron-specific depletion of CYLD-1, using two independent, neuronal RNAi-competent strains^44,45^ shortens animal lifespan (Fig. 2E and Supplementary Fig. S1E). Thus, neuronal CYLD-1 activity is necessary to sustain animal longevity.

### CYLD-1 confers neuroprotection during ageing

**Fig. 3:**
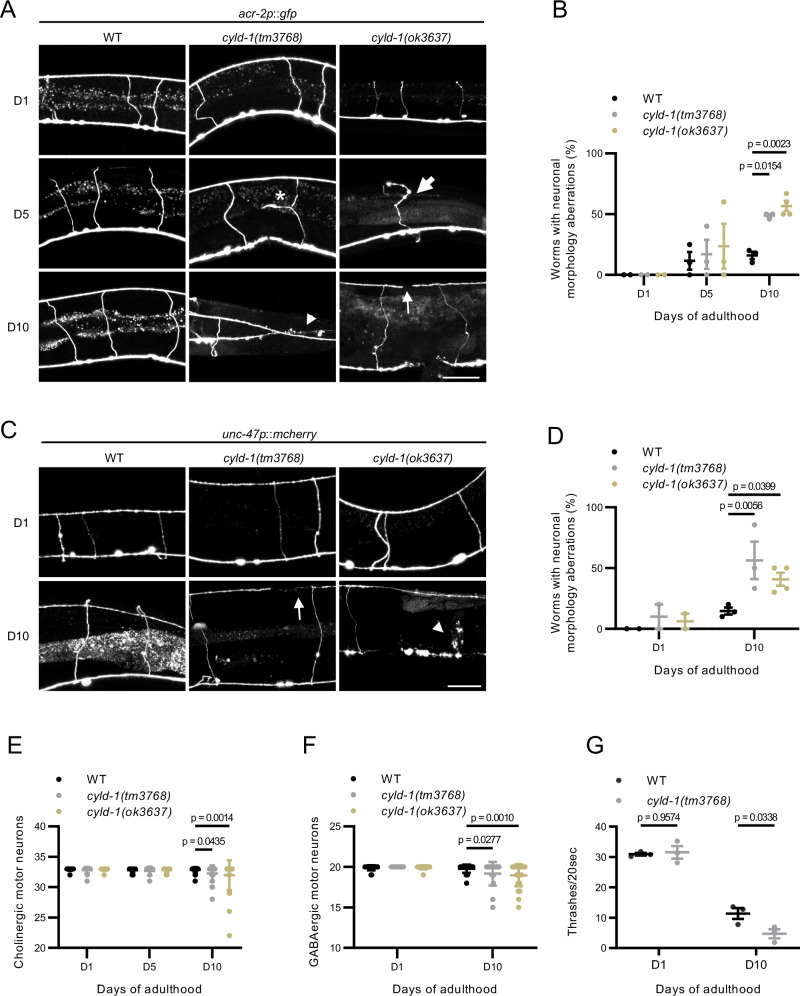
CYLD-1 protects against age-dependent motor neuron degeneration and motility decline. **A** Confocal microscopy images of D1, D5 and D10 WT, *cyld-1(tm3768)* and *cyld-1(ok3637)* worms expressing *gfp* under the control of the *acr-2* promoter, showing GFP fluorescence in A- and B-type cholinergic motor neurons. Representative neurodegeneration features are shown, including abnormal sprouting (asterisk), blebbing of neuronal commissure (thick arrow), VNC fragmentation (arrowhead) and DNC break (thin arrow). Scale bar, 50 μm. **B** Quantification of the degeneration of cholinergic motor neurons from (**A**) (*N* = 2-4 experiments, *n* ≥ 16 worms; Two-way ANOVA with two-sided Fisher’s LSD test). **C** Confocal microscopy images of D1 and D10 WT, *cyld-1(tm3768)* and *cyld-1(ok3637)* worms expressing *mcherry* under the control of the *unc-47* promoter, showing mCherry fluorescence in D-type GABAergic motor neurons. Representative neurodegeneration features are shown, including DNC break (arrow) and neuronal commissure fragmentation (arrowhead). Scale bar, 50 μm. **D** Quantification of the degeneration of GABAergic motor neurons from (**C**) (*N* = 2-4 experiments, *n* ≥ 15 worms; Two-way ANOVA with two-sided Fisher’s LSD test). **E** Quantification of the number of cell bodies of cholinergic neurons from (**A**) (*n* ≥ 16 worms from 2-4 experiments; Two-way ANOVA with two-sided Fisher’s LSD test). **F** Quantification of the number of cell bodies of GABAergic neurons from (**C**) (*n* ≥ 15 worms from 2-4 experiments, Two-way ANOVA with two-sided Fisher’s LSD test). **G** Quantification of the thrashing rate of D1 and D10 WT and *cyld-1(tm3768)* worms (*N* = 3 experiments, *n* ≥ 21 worms; Two-way ANOVA with Sidak’s multiple comparisons test). Data presented as mean ± SEM (**B**, **D**, **G**) or ± SD (**E**, **F**). Source data are provided as a Source Data file.

Alterations in neuronal function due to CYLD-1 deficiency may manifest early in life, potentially compromising animal healthspan. Hence, we examined whether CYLD-1 deficiency affects neuronal homoeostasis in young (day 1, D1) and old (day 10, D10) nematodes. As *C. elegans* ages, its nervous system undergoes neurodegeneration, marked by distinct morphological changes such as focal axon swelling, lesions in neuronal processes, and axon defasciculation^46^. In young nematodes, CYLD-1 depletion did not induce noticeable morphological abnormalities in cholinergic or GABAergic motor neurons (Fig. 3A–D, Supplementary Data 1). In sharp contrast, older *cyld-1* mutant animals exhibited exacerbated motor neuron degeneration compared to age-matched WT animals, as evidenced by defasciculation, blebbing and aberrant growth of neuronal processes, loss of commissures, as well as disruption of VNC or dorsal nerve cord (DNC) integrity (Fig. 3A–D and Supplementary Data 1). Furthermore, CYLD-1 deficiency resulted in a reduced number of surviving cholinergic and GABAergic motor neurons in aged animals (Fig. 3E, F and Supplementary Data 1).

Motor neuron dysfunction, associated with a decline in motor activity, is a hallmark of ageing in humans and other animals, including *C. elegans*^47^. Nematode locomotion in liquid, commonly referred to as thrashing, is a behavioural parameter used to assess overall motility and animal healthspan. Young *cyld-1* mutant nematodes exhibited normal motility compared to age-matched WT controls. By D10 of adulthood, *cyld-1* mutants displayed reduced thrashing rates, indicating an accelerated decline in motility, compared to age-matched WT controls (Fig. 3G). Taken together, these findings highlight the critical role of CYLD-1 in preserving motor neuron integrity and maintaining proper locomotory capacity during ageing.

### CYLD-1 preserves synaptic transmission

**Fig. 4:**
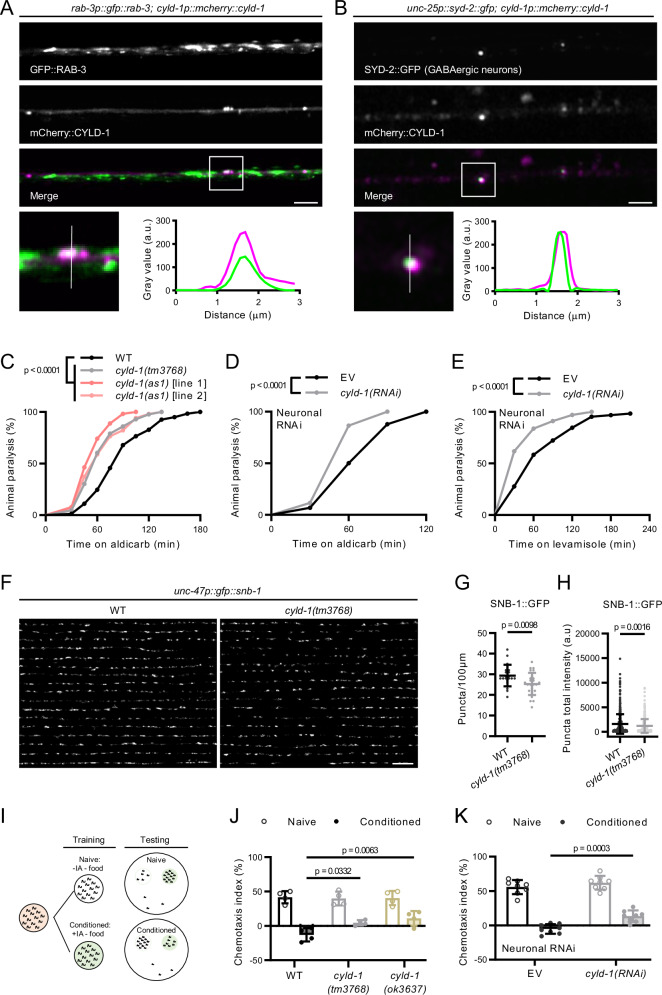
CYLD-1 promotes synaptic homoeostasis, neurotransmission and short-term associative learning. **A** Super-resolution confocal microscopy images of the VNC of D1 *rab-3p::gfp::rab-3; cyld-1p::mcherry::cyld-1* worms, showing the GFP-tagged synaptic vesicle protein RAB-3 and mCherry-tagged CYLD-1 expressed under the control of their endogenous promoters. Scale bar, 3 μm. GFP and mCherry intensity measurements correspond to the line (3μm) in the enlarged area. **B** Super-resolution confocal microscopy images of the VNC of D1 *unc-25::syd-2::gfp; cyld-1p::mcherry::cyld-1* worms, showing the GFP-tagged active zone protein SYD-2 expressed in GABAergic neurons and mCherry-tagged CYLD-1 expressed under the control of its endogenous promoter. Scale bar, 3 μm. GFP and mCherry intensity measurements correspond to the line (3μm) in the enlarged area. **C** Time-course paralysis assay on 0.5 mM aldicarb of D1 WT, *cyld-1(tm3768)* and *cyld-1(as1)* worms; log rank, Mantel-Cox test. **D** Time-course paralysis assay on 1 mM aldicarb of D1 neuronal RNAi-competent *sid-1(pk3321); unc-119p::sid-1; myo-2p::mcherry* worms treated with control (EV) or *cyld-1(RNAi)* from hatching; log rank, Mantel-Cox test. **E** Time-course paralysis assay on 0.1 mM levamisole of D1 neuronal RNAi-competent *sid-1(pk3321); unc-119p::sid-1; myo-2p::mcherry* worms treated with control (EV) or *cyld-1(RNAi)* from hatching; log rank, Mantel-Cox test. **F** Confocal microscopy images (maximum projection) of the DNC of D1 WT and *cyld-1(tm3768)* worms expressing *gfp::snb-1* under the control of the *unc-47* promoter, showing the GFP-tagged synaptic SNARE protein SNB-1 in GABAergic neurons. Scale bar, 10 μm. **G** Quantification of the number of GFP::SNB-1 positive puncta from (**F**) (*n* ≥ 23 worms from 2 experiments; Two-sided Student’s *t* test). **H** Quantification of the total intensity of GFP::SNB-1 positive puncta from (**F**) (*n* ≥ 595 puncta from at least 23 worms from 2 experiments; Two-sided Student’s *t* test). **I** Schematic illustration of the short-term associative olfactory learning protocol. Nematodes are trained for 30 min in the presence of isoamyl alcohol (IA) without food (conditioned group). Naïve worms are maintained for the same time without food in the absence of IA. Immediately after training, chemotaxis to the IA spot versus the ethanol control spot is assessed. **J** Quantification of the chemotaxis indices to IA of D1 naïve or conditioned WT, *cyld-1(tm3768)* and *cyld-1(ok3637)* worms (*N* = 4 assay plates from 2 experiments; One-way ANOVA with Dunnett’s multiple comparisons test). **K** Quantification of the chemotaxis indices to IA of D1 neuronal RNAi competent *sid-1(pk3321); unc-119p::sid-1; myo-2p::mcherry* worms, treated with control (EV) or *cyld-1(RNAi)* from hatching (*N* = 8 assay plates from 3 experiments; Two-sided Student’s *t* test). Data presented as mean ± SD. Source data are provided as a Source Data file.

Synaptic functional decline and failure are early events that precede neuronal degeneration and cell death in numerous neurodegenerative disorders^3,47^. Thus, we investigated the functional consequences of CYLD-1 depletion in the nervous system of young adult nematodes. To characterise the subcellular localisation of CYLD-1, we generated transgenic animals expressing mCherry-tagged CYLD-1 under the control of the *cyld-1* promoter (*cyld-1p::mcherry::cyld-1*). We found that CYLD-1 localizes to neuronal processes in the nerve ring, labelled by the GFP-tagged synaptic vesicle protein Ras-associated binding 3 (RAB-3)/RAB3 (Supplementary Fig. S2A). In the VNC, GFP::RAB-3 accumulates in fluorescent puncta, each corresponding to the cumulative signal from all GFP-tagged synaptic vesicles at a single chemical synapse^48^. Airyscan 2 super-resolution microscopy confirmed the synaptic enrichment of CYLD-1, which overlaps with GFP::RAB-3 in punctate structures (Fig. 4A). Furthermore, CYLD-1 colocalizes with the active zone protein SYD-2/Liprin-α, expressed under the control of the GABAergic neuron-specific promoter *unc-25* (Fig. 4B)^49^. The localization of CYLD-1 in presynaptic terminals in vivo suggests a potential role in synaptic function.

To determine whether *cyld-1* mutant nematodes exhibit synaptic deficits, we conducted pharmacological paralysis assays to assess neurotransmission efficacy. Aldicarb, a potent acetylcholinesterase inhibitor, blocks acetylcholine degradation in the synaptic cleft, causing hyperactivation of muscular acetylcholine receptors (AChRs) and eventually paralysis in *C. elegans*. Efficacy of cholinergic neurotransmission is evaluated by monitoring the time course of paralysis in the presence of aldicarb^50^. *cyld-1* mutants displayed accelerated paralysis in response to aldicarb, compared to WT animals, indicating abnormal cholinergic neurotransmission at the neuromuscular junction (NMJ) (Fig. 4C and Supplementary Fig. S2B). Notably, *cyld-1(as1)* mutants expressing catalytically inactive CYLD-1^C774S^ exhibited a similarly accelerated paralysis response compared to *cyld-1* deletion mutants upon aldicarb treatment, implicating CYLD-1 DUB activity in the regulation of cholinergic neurotransmission (Fig. 4C). The rapid paralytic effect observed in the aldicarb-sensitivity assay classifies *cyld-1* mutants as hypersensitive to inhibitors of cholinesterase (Hic), with the Hic phenotype attributed to the DUB activity of CYLD-1.

Hypersensitivity to aldicarb may result either from enhanced excitatory cholinergic neurotransmission or reduced inhibitory GABAergic neurotransmission^40,50,51^. We performed paralysis assays using levamisole, an agonist of post-synaptic levamisole AChRs (L-AChRs). Levamisole resistance is indicative of postsynaptic deficits in cholinergic NMJs, such as reduced L-AChR activity, or defective function of proteins essential for channel assembly and gating regulation^40^. We observed that *cyld-1* mutants were more sensitive than WT nematodes to levamisole (Supplementary Fig. S2C). In addition, neuron-specific depletion of CYLD-1 accelerated paralysis in response to both aldicarb and levamisole, indicating that CYLD-1 functions cell-autonomously in the nervous system to regulate neurotransmission at NMJs (Fig. 4D, E). At the NMJ, excitatory cholinergic input promotes muscle contraction, while inhibitory GABAergic input induces muscle relaxation^52^. Mutations that impair GABAergic neurotransmission, such as those that block gamma-aminobutyric acid (GABA) synthesis or loading into synaptic vesicles, result in accelerated paralysis in response to aldicarb and levamisole^51^. To investigate whether CYLD-1 depletion affects the synaptic physiology of GABAergic neurons, we monitored GABAergic synapses in the DNC using a GFP::SNB-1 (synaptobrevin 1) reporter expressed under the control of the GABAergic-specific promoter *unc-47*. Notably, *cyld-1(tm3768)* mutants had fewer synapses compared to WT nematodes (Fig. 4F, G). In addition, the total intensity of GFP::SNB-1 puncta was reduced in *cyld-1* mutant animals, indicating that each synapse contains fewer synaptic vesicles (Fig. 4F, H). To examine whether these synaptic alterations in GABAergic neurons cause perturbed GABAergic neurotransmission under CYLD-1 deficiency, we utilised pentylenetetrazole (PTZ), an antagonist of the GABA_A_ receptor. In the presence of PTZ, nematodes with underlying deficits in GABAergic neurotransmission display convulsions, reminiscent of an epileptic-like behaviour^53^. As positive controls, we used *unc-49(e407)* and *unc-43(tm1605)* mutant animals, harbouring a mutation either in the nematode GABA_A_ receptor UNC-49 or in the calcium/calmodulin-dependent protein kinase UNC-43, rendering the animals susceptible to PTZ-induced convulsions. Similar to WT nematodes, *cyld-1* mutants did not display convulsions in both solid- and liquid-based assays in the presence of different PTZ concentrations (Supplementary Fig. S2D, E). These observations suggest that *cyld-1* deficient animals preserve intact GABAergic neurotransmission despite their phenotypic alterations in GABAergic synapses (Fig. 4F–H). We hypothesise that possible GABAergic functional defects under *cyld-1* deficiency correlated with the phenotypic alterations, do not affect synaptic transmission, rendering these animals resistant to PTZ-induced epileptic-like behaviour. Therefore, we attribute the neurotransmission defects revealed by aldicarb and levamisole assays upon *cyld-1* deficiency to defective cholinergic signalling.

Coordinated synaptic transmission in neuronal circuits is essential for cognitive functions and complex behaviours, including perception, learning, and memory^54,55^. *C. elegans* exhibits both associative and non-associative learning competence, enabled by evolutionarily conserved molecular mechanisms^56,57^. To investigate whether synaptic transmission defects observed in *cyld-1* mutants are associated with cognitive impairment, we implemented an associative olfactory learning protocol to assess short-term memory. Naïve nematodes exhibit positive chemotaxis to isoamyl alcohol, a by-product of bacterial metabolism that signals the presence of food. Nevertheless, nematodes can be trained to avoid isoamyl alcohol by associating its presence with an unfavourable condition, such as the absence of food. In a two-choice behavioural test, naïve nematodes accumulate at the isoamyl alcohol spot, whereas conditioned nematodes prefer the control spot (Fig. 4I). While naïve *cyld-1* mutants and neuronally depleted *cyld-1* nematodes displayed normal chemotaxis to isoamyl alcohol (Fig. 4J, K), conditioned *cyld-1* mutants exhibited increased chemotaxis to isoamyl alcohol compared to WT animals, indicating a learning deficit (Fig. 4J). Neuron-specific depletion of *cyld-1* also impaired associative learning, further supporting the notion that CYLD-1 functions autonomously in neurons to uphold learning and memory capacity (Fig. 4K). Together, these findings suggest that CYLD-1 regulates neurotransmission underlying associative learning, in addition to excitation-inhibition balance at the NMJ.

### CYLD-1 promotes autophagic flux

**Fig. 5:**
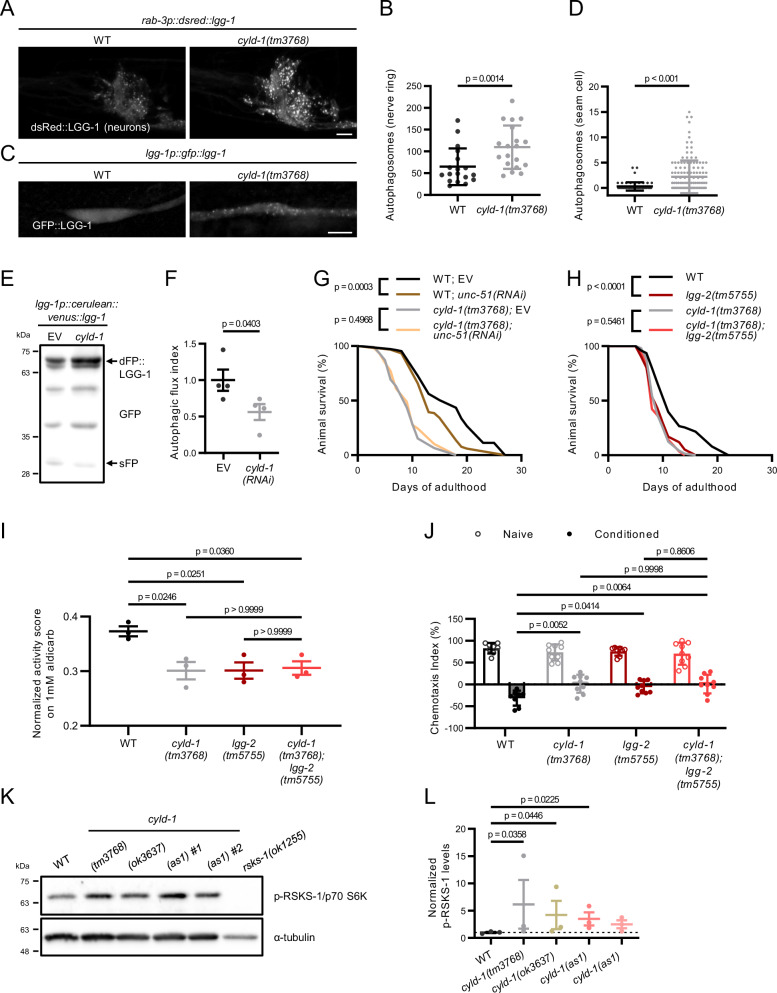
CYLD-1 preserves autophagic flux and converges with the autophagic pathway in lifespan regulation. **A** Confocal microscopy images (maximum projection) of the head area of D1 WT and *cyld-1(tm3768)* worms expressing *dsred::lgg-1* under the control of the *rab-3* promoter, depicting the dsRed-tagged autophagosomal protein LGG-1 in the nerve ring. Scale bar, 10 μm. **B** Quantification of the number of autophagosomes per nerve ring area from (**A**) (*n* ≥ 18 worms from 2 experiments; Two-sided Mann-Whitney test). **C** Widefield microscopy images of L4 WT and *cyld-1(tm3768)* worms expressing *gfp::lgg-1* under the control of the *lgg-1* promoter, showing the GFP-tagged autophagosomal protein LGG-1 in hypodermal seam cells. Scale bar, 10 μm. **D** Quantification of the number of autophagosomes per seam cell from (**C**) (*n* ≥ 69 seam cells representative of 2 experiments; Two-sided Mann-Whitney test). **E** Immunoblot analysis of lysates from D1 *eft-3p::cerulean-venus::lgg-1* worms treated with control (EV) or *cyld-1(RNAi)* from hatching, detecting dual fluorescent protein tagged LGG-1 (dFP::LGG-1) and cleaved single fluorescent protein (sFP). **F** Quantification of the sFP/dFP::LGG-1 ratio, indicative of the autophagic flux, from (**E**) (*N* = 4 experiments; Two-sided Student’s *t* test). **G** Survival curves of WT and *cyld-1(tm3768)* worms treated with control (EV) or *cyld-1(RNAi)* from hatching; log rank, Mantel-Cox test. **H** Survival curves of WT, *lgg-2(tm5755), cyld-1(tm3768) and cyld-1(tm3768); lgg-2(tm5755)* worms; log rank, Mantel-Cox test. **I** Quantification of the activity score at 60 min on 1 mM aldicarb normalised to baseline activity of WT, *cyld-1(tm3768), lgg-2(tm5755) and cyld-1(tm3768); lgg-2(tm5755)* worms (*N* = 3 experiments, each with 6-8 replicate wells containing 50–100 worms; One-way ANOVA with Tukey’s multiple comparison test). **J** Quantification of the chemotaxis indices to IA of D1 naïve or conditioned WT, *cyld-1(tm3768), lgg-2(tm5755)* and *cyld-1tm3768); lgg-2(tm5755)* worms (N ≥ 8 assay plates from 3 experiments; One-way ANOVA with Tukey’s multiple comparisons test). **K** Immunoblot analysis of lysates from D1 WT, *cyld-1(tm3768), cyld-1(ok3637)* and *cyld-1(as1)* worms, detecting endogenous p-RSKS-1/p70 S6K and α-tubulin. *rsks-1(ok1255)* mutant animals that carry a deletion altering the protein sequence after the residue 71 were used as a control for the specificity of the antibody. **L** Quantification of the p-RSKS-1 levels normalised to α-tubulin from (**K**) (*N* = 3 experiments; One-way ANOVA with Kruskal-Wallis test). Data presented as mean ± SD (**B**, **D**, **J**) or ± SEM (**F**, **I**, **L**). Source data are provided as a Source Data file.

A decline in autophagic activity has been associated with neurodegeneration in various organisms, including *C. elegans*^58–60^. K63 polyubiquitination facilitates substrate degradation in selective receptor-mediated autophagy processes, such as mitophagy, aggrephagy, and allophagy^61–64^. In addition, K63 polyubiquitination regulates autophagic flux by modifying core autophagic proteins^65,66^. To investigate the potential role of CYLD-1 in neuronal autophagy, we monitored autophagosome abundance in CYLD-1-deficient neurons, in vivo, by fluorescently tagged LGG-1, the *C. elegans* Atg8 ortholog. Depletion of CYLD-1 resulted in autophagosome accumulation in the nerve ring neurons (Fig. 5A, B). A similar increase in autophagosome numbers was observed in the hypodermal seam cells, suggesting a broader role for CYLD-1 in regulating autophagy in different tissues (Fig. 5C, D).

The accumulation of LGG-1-positive puncta could reflect either increased autophagosome formation or a blockage in later stages of autophagy. As autophagy progresses, autophagosomes fuse with lysosomes and LGG-1 is delivered into the lysosome. When LGG-1 is tagged with two fluorescent proteins (dFP::LGG-1) connected by a protease-sensitive flexible linker, a single fluorescent protein (sFP) is released by proteolytic cleavage upon delivery to the lysosome^67^. Immunoblot analysis revealed that CYLD-1 depletion reduces the sFP/dFP::LGG-1 ratio, suggesting that CYLD-1 regulates autophagic flux (Fig. 5E, F).

To test for a potential epistatic relationship between the autophagic pathway and CYLD-1, we examined the requirement for core autophagic proteins in CYLD-1-mediated lifespan regulation. Genetic suppression of autophagy, through deletion of *lgg-2/MAP1LC3* (microtubule associated protein 1 light chain), or RNAi-mediated suppression of *unc-51/unc-51 like autophagy activating kinase 1 (ULK1)*, leads to a significant reduction in *C. elegans* lifespan^68,69^ (Fig. 5G, H). Remarkably, when autophagy inhibition was combined with CYLD-1 deficiency, we observed no additive effect on lifespan shortening (Fig. 5G, H).

Inhibition of autophagy through deletion of the core autophagic gene *lgg-2*, recapitulates the defects observed upon *cyld-1* deficiency in neurotransmission and short-term associative memory of the animals (Fig. 5I, J). Furthermore, combined deletion of *cyld-1* and *lgg-2* neither exacerbates aldicarb-mediated paralysis nor aggravates compromised short-term associative memory in the chemotaxis assay towards isoamyl alcohol of *cyld-1* mutant worms (Fig. 5I, J). These findings suggest that autophagy is compromised in *cyld-1* mutant animals, and both autophagy and CYLD-1 likely function in the same pathway to promote longevity as well as preserve neuronal and cognitive function.

The master regulator of cellular metabolism, mammalian target of rapamycin (mTOR), comprises a central modulator of autophagy^70^. To examine whether this upstream negative regulator of autophagy is affected upon *cyld-1* deficiency, we assayed the activity of LET-363, the ortholog of TOR in *C. elegans*^71^, by monitoring the S386 phosphorylation of its substrate RSKS-1^72,73^. This phosphorylation corresponds to the conserved S371 of the mammalian p70-S6 Kinase. *cyld-1* mutant animals, including the endogenous catalytically inactive ones, exhibited increased phospho-RSKS-1 levels compared to WT worms, suggesting that *cyld-1* deficiency leads to LET-363/mTOR signalling activation (Fig. 5K, L).

### CYLD-1 modulates autolysosomal and lysosomal morphology

**Fig. 6:**
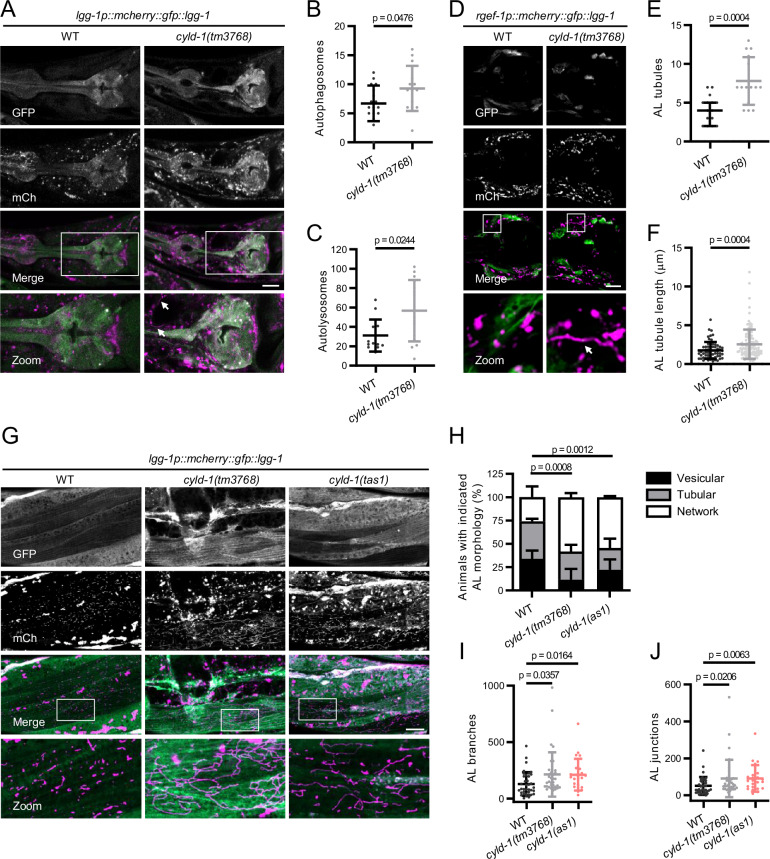
CYLD-1 regulates autolysosomal dynamics and function. **A** Confocal microscopy images of the head area of D1 WT and *cyld-1(tm3768)* worms expressing *mcherry::gfp::lgg-1* under the control of the *lgg-1* promoter, showing the mCherry-GFP-tagged autophagosomal protein LGG-1 in the pharynx and nerve ring area. Autophagosomes appear as GFP and mCherry positive structures, while autolysosomes appear as mCherry only positive structures. Arrows indicate elongated autolysosomal structures present in the *cyld-1(tm3768)* worms. Scale bar, 10 μm. **B** Quantification of the number of autophagosomes in the posterior pharyngeal bulb from (**A**) (*n* ≥ 14 worms from 3 experiments; Two-sided Student’s *t* test). **C** Quantification of the number of autolysosomes in the posterior pharyngeal bulb from (**A**) (*n* ≥ 13 worms from 3 experiments; Two-sided Mann-Whitney test). **D** Super-resolution confocal microscopy images of the head area of D1 WT and *cyld-1(tm3768)* worms expressing *mcherry::gfp::lgg-1* under the control of the *rgef-1* promoter, showing the mCherry-GFP-tagged autophagosomal protein LGG-1 in neuronal cell bodies and processes of the nerve ring. Arrow indicates an elongated autolysosomal structure present in the *cyld-1(tm3768)* worms. Scale bar, 10 μm. **E** Quantification of the number of autolysosomal tubules per nerve ring area from (**C**) (*n* = 15 worms from 2 experiments; Two-sided Student’s *t* test). **F** Quantification of the autolysosomal tubule length from (**C**) (n ≥ 62 tubules from 15 worms from 2 experiments; Two-sided Mann-Whitney test). **G** Super-resolution confocal microscopy images of the mid-body area of D1 WT, *cyld-1(tm3768)* and *cyld-1(as1)* worms expressing *mcherry::gfp::lgg-1* under the control of the *lgg-1* promoter, showing the mCherry-GFP-tagged autophagosomal protein LGG-1 in the hypodermis (hyp7 and seam cells) and body wall muscle cells. Scale bar, 10 μm. **H** Quantification of the autolysosomal morphology from (**G**) (*n* ≥ 21 worms from 2 experiments; Two-sided chi-square test). **I** Quantification of the number of autolysosomal skeleton branches per hypodermal area from (**G**) (*n* ≥ 23 worms from 2-4 experiments; Kruskal-Wallis with Dunn’s multiple comparison test).**J** Quantification of the number of autolysosomal skeleton junctions per hypodermal area from (**G**) (*n* ≥ 23 worms from 2-4 experiments; Kruskal-Wallis with Dunn’s multiple comparison test). Data presented as mean ± SD (**B**, **C**, **E**, **F**, **I**, **J**) or ± SEM (**H**). Source data are provided as a Source Data file.

To evaluate potential differences in the abundance of autophagic vesicles before and after fusion with lysosomes, we utilised the tandem mCherry::GFP::LGG-1 reporter strain, in which expression is driven by the endogenous *lgg-1* promoter^74^. Autophagosomes appear as both GFP- and mCherry-positive structures. Upon autophagosome-lysosome fusion, autolysosomes appear as GFP-negative and mCherry-positive structures, since the acidic lysosomal pH quenches GFP fluorescence. The *cyld-1(tm3768)* mutants exhibited a systemic increase in mCherry fluorescence, indicating a widespread accumulation of autolysosomes (Supplementary Fig. S3A, B). An increased number of both autophagosomes and autolysosomes was observed in the pharynx area of *cyld-1* mutants compared to WT animals (Fig. 6A–C). Notably, autolysosomes in *cyld-1* mutants adopted an elongated tubular structure in the nerve ring area, in contrast to the predominantly vesicular autolysosomes seen in WT nematodes (Fig. 6A, arrows in the zoomed area). We confirmed this distinctive morphology using neuron-specific expression of the mCherry::GFP::LGG-1 reporter (Fig. 6D). Morphometric analysis of neuronal autolysosomes revealed that CYLD-1 deficiency is associated with a significant increase in both the number and length of autolysosomal tubules (Fig. 6D–F). A similar effect was observed in the hypodermis of *cyld-1* deletion and catalytically inactive mutants, where autolysosomes formed elaborate networks (Fig. 6G, H). *cyld-1* mutants also exhibited an elevated number of autolysosomal skeleton branches and junctions and increased skeleton length, indicating that autolysosomes form complex, interconnected networks (Fig. 6I, J and Supplementary Fig. S3C–F).

**Fig. 7:**
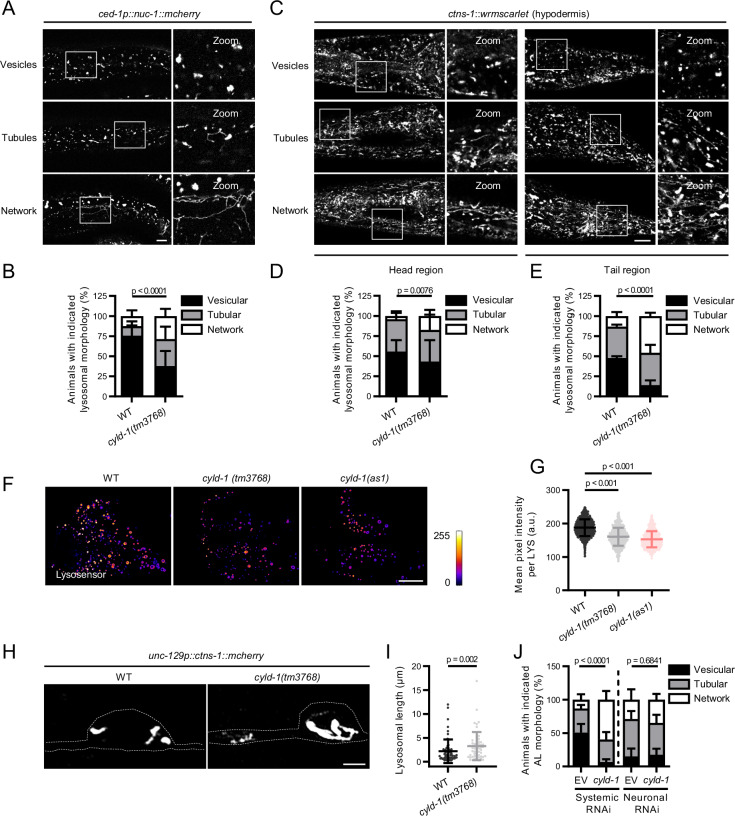
CYLD-1 regulates lysosomal network morphology and function. **A** Confocal microscopy images of the posterior body area of D1 worms expressing *nuc-1::mcherry* under the control of the *ced-1* promoter, monitoring the mCherry-tagged lysosomal nuclease NUC-1 in hypodermis. Lysosomes are classified based on their morphology as vesicles, short tubules and tubular networks. Scale bar, 10 μm. **B** Quantification of the lysosomal morphology of D1 WT and *cyld-1(tm3768)* worms expressing *nuc-1::mcherry* under the control of the *ced-1* promoter from (**A**) (*n* ≥ 38 worms from 3 experiments; Two-sided chi-square test). **C** Super-resolution confocal microscopy images of the head and tail area of D1 worms expressing endogenously tagged *ctns-1::wrmscarlet*, showing the wrmScarlet-tagged lysosomal membrane marker CTNS-1 in hypodermis. Lysosomes are classified based on their morphology as vesicles, short tubules and tubular networks. Scale bar, 10 μm. **D** Quantification of the lysosomal morphology at the head area of D1 WT and *cyld-1(tm3768)* worms expressing endogenously tagged *ctns-1::wrmscarlet* from (**C**) (*n *= 22 worms from 2 experiments; Two-sided chi-square test). **E** Quantification of the lysosomal morphology at the tail area of D1 WT and *cyld-1(tm3768)* worms expressing endogenously tagged *ctns-1::wrmscarlet* from (**C**) (*n* ≥ 22 worms from 2 experiments; Two-sided chi-square test). **F** Confocal microscopy images of D1 WT, *cyld-1(tm3768)* and *cyld-1(as1)* worms stained with LSG, showing lysosomes in the proximal intestinal cells. Images are pseudo-coloured using the FIJI Fire LUT. Scale bar, 10 μm. **G** Quantification of the mean pixel intensity per lysosome from (**F**) (n ≥ 785 lysosomes from 11–13 worms from 2 experiments; Kruskal-Wallis test with Dunn’s multiple comparisons test). **H** Super-resolution confocal microscopy images of D1 WT and *cyld-1(tm3768)* worms expressing *ctns-1::mcherry* under the control of the *unc-129* promoter, showing the mCherry tagged lysosomal membrane protein CTNS-1 in the first visible cholinergic neuron of the VNC. Dashed lines denote the cell boundaries. Scale bar, 2 μm. **I** Quantification of the lysosomal tubule length from (**H**) (*n* ≥ 53 lysosomes from 17–20 neurons from 3 experiments; Two-sided Mann-Whitney test). **J** Quantification of the autolysosomal morphology from D1 worms expressing *mcherry::gfp::lgg-1* under the control of the *lgg-1* promoter, upon systemic or neuronal-restricted *cyld-1(RNAi)* (n ≥ 32 worms from 4-5 experiments; Two-sided chi-square test). Data presented as mean ± SEM (**B**, **D**,** E**, **J**) or ± SD (**G**, **I**). Source data are provided as a Source Data file.

During ageing, lysosomal morphology in *C. elegans* becomes disrupted, with lysosomes transitioning from their typical vesicular structure to a tubular network in the hypodermis^75^. These morphological changes are accompanied by functional impairment, including reduced lysosomal motility, acidity, and degradative capacity^75^. The autolysosomal morphology in *cyld-1* mutant animals that we observed recapitulates age-related lysosomal phenotypes. To validate the lysosomal nature of the network, we used transgenic nematodes expressing the lysosomal nuclease NUC-1, fused with mCherry, under the control of the hypodermal promoter *ced-1*^75^. Indeed, prominent lysosomal networks were formed in *cyld-1* mutants (Fig. 7A, B). Morphometric analysis revealed an increased lysosomal skeleton size, as well as an increased number of lysosomal branches and junctions on D1 of adulthood compared to WT animals (Supplementary Fig. S3G–I). Similarly, c*yld-1* deficient animals expressing the lysosomal membrane marker CTNS-1 endogenously tagged with wrmScarlet revealed increased lysosomal network formation in the hypodermis (Fig. 7C–E). In addition, we examined lysosomal acidity by staining live nematodes with LysoSensor Green (LSG), a fluorescent probe that accumulates in acidic organelles and shows a pH-dependent increase in fluorescence intensity. In *cyld-1* mutants, intestinal stained structures exhibited reduced fluorescence, indicating the presence of less acidic organelles (Fig. 7F, G). Notably, CYLD-1^C774S^ animals showed impaired lysosomal acidification as well, underscoring the critical role of CYLD-1 catalytic function in maintaining lysosomal homoeostasis (Fig. 7F, G). Collectively, these findings suggest that CYLD-1 promotes autophagy and preserves lysosomal homoeostasis in various tissues of *C. elegans*.

Since CYLD-1 specifically supports cholinergic neurotransmission, we investigated whether CYLD-1 impacts lysosomal architecture in this neuronal subpopulation. c*yld-1* deficient animals expressing the lysosomal membrane marker CTNS-1::mCherry in cholinergic neurons of the VNC exhibited elongated lysosomes compared to WT animals (Fig. 7H, I), consistent with the previous results concerning the autolysosomal phenotype upon *cyld-1* deficiency in neurons of the nerve ring (Fig. 6D–F). To examine whether neuronal CYLD-1 cell non-autonomously affects the autophagic-lysosome system in hypodermis, where similar lysosomal architecture is observed upon *cyld-1* deficiency, we compared the impact of systemic to neuronal-specific RNAi-mediated depletion of CYLD-1 in autolysosomal morphology of hypodermis. Systemic depletion of CYLD-1 results in increased autolysosomal network formation in the hypodermis, while neuronal-specific depletion of CYLD-1 does not affect the architecture of hypodermal autolysosomes (Fig. 7J). Overall, these results indicate that neuronal CYLD-1 regulates the autophagic-lysosome system cell-autonomously, without cell non-autonomous functions in hypodermal lysosome physiology.

## Discussion

Our study reveals a central role of CYLD-1 in the regulation of ageing in *C. elegans*. We further delineate the involvement of CYLD-1 activity in maintaining neuronal physiology, as well as behavioural outcomes during ageing. In young nematodes, CYLD-1 maintains synaptic homoeostasis and neurotransmission through its catalytic DUB activity. When challenged, CYLD-1 deficient animals show impaired associative short-term memory. In older animals, CYLD-1 preserves motor neuron homoeostasis, sustaining locomotory capacity. CYLD-1 supports catabolic processes associated with the autophagy-lysosomal pathway in both neurons and peripheral tissues, while its deficiency induces prominent alterations to the architecture of the autophagosomal and lysosomal network.

Genome-wide association studies have identified CYLD mutations in patients with neurodegenerative disorders, including frontotemporal dementia, amyotrophic lateral sclerosis, and Alzheimer’s disease^29–31,76^. However, these studies do not consistently show an enrichment of CYLD variants among patients. Moreover, functional studies to confirm the pathogenicity of these CYLD variants are lacking^31^. Distinct CYLD structural and functional elements, such as three N-terminal CAP-Gly domains and a C-terminal catalytic USP domain, confer a range of molecular functions. The first two CAP-Gly domains facilitate microtubule binding, while the third binds to NEMO/IKKγ, the regulatory subunit of the IKK complex^20,37,38^. CYLD and A20/TNFα-induced protein 3 (TNFAIP3), two deubiquitinases with diverse structural domains, have largely overlapping substrate repertoire^77^. However, mice deficient in either enzyme exhibit significant phenotypic differences, indicating non-redundant functions and substrate selectivity^77^.

Here, we report that CYLD-1 is essential for preserving both physiological and extended lifespan in *C. elegans*. We further show that a single point mutation in the catalytic cysteine recapitulates the reduced lifespan observed in *cyld-1*-ablated animals, demonstrating that CYLD-1 DUB activity is crucial for longevity. This deubiquitinase activity is indispensable and non-redundant in vivo. Interestingly, neuron-specific CYLD-1 depletion shortens animal lifespan. During ageing, CYLD-1 preserves motor neuron integrity, supporting intact locomotory aptitude. By contrast, CYLD-1 deficiency leads to axonal degeneration, loss of cholinergic and GABAergic neurons, and impaired locomotion.

Research in mice and primary neuronal cultures indicates that Cyld regulates synaptic homeostasis^25,26^. Alterations in neurotransmission and neuronal excitability have been observed, alongside synaptic plasticity impairment, including long-term potentiation and depression^24–27,78^. A range of cognitive deficits have been linked to Cyld deficiency,such as impaired fear memory^27^, autism spectrum-like disorders^26^ and anxiety-like^25^ behaviour in mice. However, there are discrepancies relevant to the Cyld mode of action, in functional and behavioural reports. One study in *Cyld* knockout mice documented a reduction in synaptic spines in CA1 hippocampal neurons, with normal motor function and anxiety-like behaviour^26^. By contrast, another study reported increased synaptic spine numbers in CA1 neurons, impaired locomotion, and increased anxiety-like behaviour^25^.

Our results show that CYLD-1 supports cholinergic neurotransmission and GABAergic synapse integrity. We found that young *cyld-1* mutant animals exhibit increased susceptibility to cholinesterase inhibition, or activation of postsynaptic AChRs. These perturbations lead to overactivation of AChRs, muscle hypercontraction, and eventually paralysis^50,79^. Specifically, we observed that CYLD-1 DUB activity protects against the development of induced paralysis. Moreover, animals with neuron-specific CYLD-1 downregulation show enhanced sensitivity to pharmacologically-induced paralysis. CYLD-1 deficiency also leads to decreased number of GABAergic synapses and synaptic vesicle content in GABAergic neurons. Nevertheless, GABAergic neurotransmission is intact in these animals. The *C. elegans* genome encodes a diverse array of ionotropic AChRs, with tissue-specific expression in muscles and the nervous system^80^. In muscle cells, two classes of excitatory AChRs exist, each with distinct pharmacological properties: levamisole-sensitive AChRs and nicotine-sensitive AChRs. Neurons express a distinct class of AChRs with neuro-specific subunits that are insensitive to levamisole^81^. The lack of *cyld-1* expression in body wall muscle cells, combined with accelerated paralysis in response to levamisole following neuronal CYLD-1 depletion, suggests that CYLD-1 regulates cholinergic synapses between neurons, or the presynaptic cholinergic domains of NMJs^82^.

Our study also highlights a critical role for CYLD-1 in maintaining lysosomal morphology and acidity. To our knowledge, this is the first study linking CYLD deficiency to lysosomal dysfunction. K63 polyubiquitination has been implicated in autophagy regulation and cargo selectivity^9,83^. We show that CYLD-1 regulates autophagosomal and lysosomal physiology in neurons in a cell-autonomous manner and peripheral tissues in vivo. CYLD-1 and autophagy converge to maintain lifespan in *C. elegans*. CYLD-1 downregulation inhibits autophagic flux, causing accumulation of autolysosomal and lysosomal tubules. We hypothesize that disruption of CYLD-1 DUB activity leads to stabilisation of K63 polyubiquitin chains on substrates, which may include autophagic cargo marked for degradation^84^ and / or key autophagic components, such as Ulk1^66^, or Beclin1^85^. Members of the USP family of DUBs, including USP1, USP14, and A20, have been shown to modulate autophagy by deubiquitinating these proteins in mammalian cells^86^. CYLD also interacts with the mammalian target of rapamycin (mTOR), an upstream autophagy inhibitor, further suggesting a role for CYLD in autophagy regulation^26^. Previous studies have demonstrated that mTOR activity is regulated by TRAF6-mediated K63 polyubiquitination during nutrient starvation, leading to autophagy inhibition^87^. Our results demonstrate increased mTOR activity upon *cyld-1* deficiency. Therefore, mTOR is a potential CYLD substrate, implicated in autophagy regulation. It is also possible that CYLD promotes autophagy in a DUB-independent manner; e.g. by inhibiting HDAC6, which promotes autophagosome-lysosome fusion via cortactin deacetylation^20,88,89^.

In conclusion, our findings highlight the importance of fine-tuning neuronal lysine63 polyubiquitination in preserving neural activity and cognitive functions, optimizing organismal fitness and enhancing survival. Identification of specific CYLD-1- DUB substrates may generate new molecular insights into synaptic function and provide therapeutic targets for age-related neurodegenerative pathologies impacting locomotion and cognition.

## Methods

### *C. elegans* strains and genetics

We followed standard procedures for maintaining *C. elegans* strains. Rearing temperature was set to 20 °C unless noted otherwise. Nematodes were cultured on nematode growth medium (NGM) streptomycin-supplemented plates, seeded with OP50 *E. coli* bacteria. For RNAi experiments, nematodes were cultured on NGM ampicillin-supplemented plates, seeded with HT115 *E. coli* bacteria expressing RNAi constructs or the empty vector pL4440. IPTG was added to the liquid cultures at a final concentration of 2 mM prior to seeding. Bacterial clones expressing RNAi constructs were obtained from the Ahringer library (*cyld-1*, *III-5G02*) or the Tavernarakis laboratory collection (*unc-51*, p281;* daf-2*, p334 ). Nematodes were treated with dsRNA expressing bacteria from embryos unless noted otherwise. The full list of strains used in this study is provided in Supplementary Table 1.

### Cloning

To generate the transcriptional reporter of *cyld-1*, the 1.5 kb genomic region upstream of the *cyld-1* ATG start codon was isolated from genomic DNA, using *cyld-1p* forward (FW) and reverse (RV) primers containing AgeI and XbaI sites, respectively and subsequently inserted in the modified plasmid pPD117.01 (L3691), where the original GFP sequence was replaced with mCherry (p2880). The resulting plasmid (p2899) was validated through colony PCR and digestions with restriction enzymes. To generate the translational reporter of *cyld-1*, *C. elegans* total RNA was used as a template to synthesise *cyld-1* cDNA using the PrimeScript™ Reverse Transcriptase kit (Taqara) and the *cyld-1* full coding sequence (CDS) RV primer. *cyld-1* cDNA was subsequently amplified using Expand High Fidelity (Roche) polymerase, using the *cyld-1* full CDS FW and RV primers containing NheI sites. The produced 3.5 kb fragment was inserted in the TOPO vector, and the resulting plasmid (p2900) was validated through SspI/XbaI digestion. From the plasmid p2900, *cyld-*1 full CDS was isolated as an NheI cassette and subsequently inserted into the plasmid p2899 linearised with NheI, downstream of the mCherry sequence to achieve an N-terminal fusion (*cyld-1p::mcherry::cyld-1)*. The resulting plasmid (p3102) was validated through colony PCR, digestions with restriction enzymes and sequencing.

To generate the neuronal HA tagged ubiquitin plasmids, *rab-3p* was isolated from the plasmid p1915, using SphI/BamHI digestion. The 2056 bp fragment corresponding to *rab-3p* was inserted into the plasmid pPD117.01 (L3691), where *mec-7p* sequence was removed using SphI/BamHI digestion. The resulting plasmid *rab-3p::gfp* (p3110) was validated through colony PCR and EcoRI digestion. HA::Ubiquitin^WT^ and HA::Ubiquitin^K63^ were amplified with PCR from Addgene plasmids 17608 and 17606, using HA FW and Ub RV primers, containing restriction sites for KpnI and NheI enzymes, respectively. The resulting 312 bp PCR products were purified, digested with KpnI/NheI, and inserted into the plasmid p3110, where the GFP sequence was removed through KpnI/NheI digestion. The resulting plasmids *rab-3p::ha::ub*^*WT*^ (p3113) and *rab-3p::ha::ub*^*K63*^ (p3114) were validated through digestions with restriction enzymes. All primers are listed in Supplementary Table 2.

### Nematode strain generation

New strains were generated using biolistic bombardment (PDS-1000/He system, BioRad) of nematodes with the *unc-119(ed3)* genetic background. 2-3 μg of selected plasmid DNA and an equal amount of *unc-119* rescue plasmid DNA were used. In order to facilitate subsequent crossing of the generated nematode strains, in some cases, an additional fluorescent co-transformation marker was used (pPD112.11 for the expression *myo-2p::gfp*).

### CRISPR-Cas mutagenesis

To generate the catalytically inactive CYLD-1 variant, we performed CRISPR mutagenesis to substitute catalytic cysteine774 with serine, following standard protocols^90,91^. Single guide RNA (sgRNA) consisted of a tracer RNA (tracrRNA) sequence with catalytic activity and a designated CRISPR RNA (crRNA) sequence targeting the *cyld-1* locus near the C774. A 200 bp homology repair (HR) template flanking the mutation site introduced the desired edit TGT→TCT as well as two silent mutations creating a new XbaI site and destroying an existing EcoRI site to facilitate selection of mutagenized lines. crRNA and HR template were designed using the Benchling CRISPR design tool (Benchling [Biology Software]. (2026). Retrieved from https://benchling.com). tracrRNA, crRNA and HR template were ordered by IDT as RNAs and single stranded oligodeoxynucleotide (ssODN), respectively. A parallel co-CRISPR mutagenesis strategy targeting the *dpy-10* locus was implemented to allow initial phenotypic screening of CRISPR success. All oligonucleotide sequences are provided in Supplementary Table 2.

An injection mix consisting of 25 mM KCl, 7.5 mM HEPES pH 7.4, 200 ng/μL tracrRNA, 150 ng/μL *dpy-10* crRNA, 13.75 ng/μL *dpy-10* ssODN, 300 ng/μL *cyld-1*^*C774S*^ crRNA, 100 ng/μL *cyld-1*^*C774S*^ ssODN and 2.6 μΜ Cas9 (M0386T, NEB) was prepared and incubated at 37 °C for 15 min. Microinjections to *C. elegans* gonads were performed using standard techniques^92^. Injected nematodes were allowed to recover at 25 °C overnight and subsequently shifted to 20 °C. F1 progeny with roller or dumpy phenotypes, corresponding to *dpy-10(cn64)*/+ or *dpy-10(0),* respectively, were picked, and one worm per plate was allowed to give progeny. To identify potential successful *cyld-1*^*C774S*^ lines, we isolated genomic DNA and performed digestions of PCR amplified regions (primer set *tm3768* FW and RV) using EcoRI and XbaI restriction enzymes. Positive lines were subsequently validated with sequencing. Two independent lines bearing the desired mutation were isolated and outcrossed three times to the WT N2 strain to remove *dpy-10(cn64)* and additional potential off-target mutations. The newly generated variation was named *cyld-1(as1)*.

### Assessment of associative olfactory learning

Synchronised young adult nematodes were collected from plates and thoroughly washed four times with M9 solution (51 mM NaCl, 37 mM KH_2_PO4, 44 mM Na_2_HPO_4_, 1.7 mM MgSO_4_) to remove bacteria. Nematodes were allowed to settle by gravity between washes. Worms were divided in naïve and conditioned populations. Naïve worms were transferred on 60 mm plates without food, sealed with parafilm and stored at room temperature. Conditioned worms were transferred on 60 mm plates, with 3 μL isoamyl alcohol placed on the lid, sealed with parafilm and stored at room temperature. The conditioning period was 90 min. During this period, assay plates were prepared. To this end, 15 μL of 200 mM sodium azide were spotted on 10 cm NGM plates, on the designated spot and counter-spot and allowed to be absorbed for 15 min. 3 μL of 1:1000 diluted isoamyl alcohol in 100% ethanol was spotted on a small parafilm piece on top of the designated spot. Similarly, 3 μL 100% ethanol were spotted on a small parafilm piece on top of the designated counter-spot. Upon completion of the conditioning period, naïve and conditioned worms were separately collected using M9 solution, washed and spotted on the starting spot on the assay plates. Nematodes were allowed to freely move for 2 h at room temperature, and afterwards, chemotaxis was manually evaluated. Chemotaxis index (CI) was calculated as the number of worms on the isoamyl alcohol spot minus the number of worms on the counter-spot, divided by the total number of worms (%CI = 100 ns (IA-C) / total). Learning index was calculated as the CI_naïve_ - CI_conditioned_. 50-150 worms were used per assay plate.

### Motility assays (thrashing)

Synchronised nematodes of the indicated age were assessed for their motility in M9 solution. 40 μL of M9 solution were placed in one well of a 96-well plate, and one nematode was immersed in each well. The number of body bends per 20 sec was manually scored under a dissecting stereoscope.

### Pharmacological paralysis assays

Aldicarb and levamisole paralysis assays on plates were performed as previously described^50,53^. Freshly made NGM plates were supplemented with the indicated concentration of aldicarb (33386, Sigma-Aldrich) or tetramisole hydrochloride (L9756, Sigma-Aldrich). Plates were incubated 1 h at room temperature for drug absorption and subsequently spotted with 50 μL OP50 bacteria culture. For each replicate, 2-3 assay plates containing 15–40 animals were assayed per experimental condition. Animals were assessed for provoked movement every 20 min.

Aldicarb paralysis assays in liquid were performed using the WMicrotracker ONE device (Phylumtech S.A., Santa Fe, Argentina), which measures movement via infrared light scattering in a 96-well plate format. Animals were collected from plates with M9 supplemented with 10% OP50 bacteria culture and distributed in 96-well plates with approximately 50–100 animals per well and 6-8 technical replicates per condition. Basal activity was recorded for 30 min and used for normalisation. Subsequently, aldicarb was added to each well to a final concentration of 1 mM, and well activity was monitored and analysed at 60 min.

Pentylenetetrasole- (PTZ-)induced epileptic seizure-like activity was analysed on plate and liquid, as previously described^53,93^. For the plate-based assay, PTZ (P6500, Sigma-Aldrich) was added on plate at 10 mg/mL, and plates were incubated for 1 h at room temperature for drug absorption. 10–20 animals were scored for convulsions every 30 min. For the liquid-based assay, 10–15 animals were placed in a 50 μL drop of 5 or 10 mg/mL PTZ diluted in M9. Animals were scored for convulsions every 15 min.

### Lifespan assays

Lifespan assays were performed at 20 °C, with the exception of *glp-1(e2141ts)* mutants that were raised at the restrictive temperature (25 °C) for 48 h from egg stage and then shifted to 20 °C. Synchronous animal populations were generated by hypochlorite treatment, timed egg-laying of hermaphrodite mothers or synchronisation at the L4 stage. 20–50 worms were placed in a 60 mm plate and were transferred daily or every second day on fresh plates to avoid contamination with progeny. Upon completion of the reproductive period, worms were transferred every two to three days. Survival was scored every one to three days, based on provoked movement. Missing worms and worms with internally hatched eggs or protruding vulvas were censored from the analysis. Lifespan experiments were performed at least twice. Analysis was performed using the Log-rank (Mantel-Cox) test. A full list of lifespan statistics is provided in Supplementary Table 3.

### Lysosomal staining

LysoSensor™ Green DND-189 (L7535, Invitrogen) was used to stain acidic lysosomes. Synchronised L4 nematodes were transferred on UV-inactivated OP50 seeded NGM plates, containing 1 μΜ LSG and incubated overnight at 20 °C in the dark. The next day, D1 adult worms were transferred on NGM OP50 seeded plates without dye to allow removal of excess dye. An unstained negative control sample was used to ensure signal specificity.

### Microscopy and image analysis

Worms were immobilised on 2% agarose pads using 10 mM tetramisole, or 10 mM sodium azide for the experiments evaluating LGG-1 fluorescent markers, in accordance with the current guidelines for monitoring autophagy in *C. elegans*^94^. For epifluorescence microscopy, we used the AxioImager Z2 epifluorescence microscope (Zeiss) or EVOS FV Auto 2 Imaging system (Thermofisher Scientific). For confocal microscopy, we used either an SP8 confocal microscope (Leica) or an LSM900 microscope (Zeiss). For super-resolution microscopy, we used the LSM900 microscope equipped with an Airyscan 2 detector (Zeiss).

GFP::LGG-1 puncta number in seam cells was counted directly under an epifluorescent microscope, and representative images of seam cells were obtained (strains DA2123, IR2846). Cholinergic and GABAergic neuronal integrity and cell number in the VNC and tail were evaluated directly under the confocal microscope, and representative images of degenerated neuronal processes were obtained (strains CZ631, IR2963, IR3373, IR2375, IR2964, IR3372). Assessment of morphological aberrations associated with ageing, includes VNC and DNC fragmentation and defasciculation, missing or fragmented commissures and blebbing of neuronal processes^95–99^.

All other analyses were performed using the FIJI software (version 2.14.0/1.54p)^100^. For the analysis of mCherry::GFP::LGG-1 puncta in the posterior pharyngeal bulb, single optical slices were obtained using a Leica SP8 confocal microscope (strains MAH215 and IR3069). For the analysis of dsRed::LGG-1 puncta in the nerve ring, maximum projection images of nerve rings were obtained using a Leica SP8 confocal microscope (strains IR2379, IR3150). For the analysis of NUC-1::mCherry-marked lysosomes, single optical slices of the posterior body area were obtained using a Leica SP8 confocal microscope (stains XW5399, IR3164). For the analysis of mCherry::GFP::LGG-1-marked autolysosomes in the hypodermis and nerve ring neurons (strains MAH215, IR3069, IR3293, MAH508, IR3071), CTNS-1::WrmScarlet-marked lysosomes in the hypodermis (strains PHX5270 and IR3367) and CTNS-1::mCherry-marked lysosomes in cholinergic neurons (strains KG2430 and IR3264), single optical slices were obtained using an LSM900 confocal microscope equipped with an Airyscan 2 detector. For the analysis of mCherry::CYLD-1 co-localisation with synaptic proteins, single optical slices of posterior VNCs were obtained using an LSM900 confocal microscope equipped with an Airyscan 2 detector (strains IR3072, IR3073). Super-resolution processed images were produced using the ZEN Blue software. Intensity plots of single pixel values along a single pixel line crossing the synapse signal were generated using FIJI. For the analysis of LSG-stained particles, single optical slices of the proximal intestine were obtained using an LSM900 confocal microscope.

Image processing and segmentation was performed with FIJI software [subtract background, enhance local contrast (CLAHE), denoise] and threshold values were used to obtain binary images. For the morphometric analysis of neuronal autolysosomes, binary images were used for particle analysis. The following parameters were established as descriptors of neuronal autolysosome tubules (size > 0.001 μm, circularity < 0.7, aspect ratio > 3). The resulting particles corresponding to autolysosome tubules were manually validated to exclude particles corresponding to fused autolysosome vesicles or out of focus signal. Autolysosome tubule particles were converted to skeletons using the skeletonise function of FIJI software, and their length was measured using the longest shortest path measurement of analyse skeleton function^101^. For the quantitative analysis of autolysosome and lysosome networks, binary images were converted to skeletons using the skeletonise function of the FIJI software. Skeletons were analysed using the analyse skeleton function to measure the number of junctions and branches per skeleton. Total skeleton length was calculated as the sum of slab, endpoint and junction pixels. For the quantitative analysis of neuronal lysosome length in cholinergic neurons, lysosomes were manually traced. For qualitative analysis of autolysosomal and lysosomal morphology, autolysosomes/lysosomes were classified as vesicles, (short) tubules or tubular networks by a blinded experimenter.

For the analysis of LSG positive lysosomes, binary images were analysed with the MorphoLibJ plugin in FIJI (2D opening size = 10) to select individual lysosomes as regions of interest (ROIs). The multi-measure function of ROI Manager was used in the original image to measure the mean pixel intensity per lysosome.

### Immunoblotting

Large populations of synchronised animals of the indicated age were collected, and washed thoroughly with M9 solution to remove bacteria. Worm pellets were snap-frozen in liquid nitrogen. Pellets were mechanically lysed with the 0.5 mm zirconium oxide beads (ZrOB05, Next Advance) using the Bullet Blender^®^ (Next Advance) homogeniser in lysis buffer (50 mM Tris pH7.4, 150 mM NaCl, 1 mM EDTA). Protein lysates were supplemented with detergents (1% Triton X-100, 0.5% sodium deoxycholate, 0.1% SDS) and incubated for 10 min shaking at 4 °C. Lysates were centrifuged for 10 min at 17000 × *g*, and supernatants were stored at −20 °C. Protein quantification was performed using Pierce^™^ BCA Protein Assay Reagent A kit (23225, ThermoFisher Scientific). 5–80 μg of total protein was used for polyacrylamide gel electrophoresis with Tris-Glycine or Bis-Tris/MOPS gel system. Proteins were transferred to Amersham Protran 0.2 NC nitrocellulose Western blotting membrane (10600001, Cytiva). Blocking was performed with blocking buffer (5% non-fat skim milk or 5% BSA diluted in 0.1% TBS-T). The following primary antibodies were used diluted in 3-5% blocking buffer and supplemented with 0.02% sodium azide (S2002, Sigma-Aldrich): GFP (1:10000; 701, Minotech), HA (clone C29F4; 1:1000; 3724, Cell Signalling), Ubiquitin Antibody Lys63-Specific (clone Apu3; 1:1000; 05-1308, Merck), α-tubulin (1:10000; 12G10, DSHB), p-p70 S6 Kinase (Ser371) (1:1000; 9208, Cell Signalling). The following secondary antibodies were used, diluted 1:10000 in 3% blocking buffer: Goat anti-Mouse HRP (ab6789, Abcam), Donkey anti-Rabbit HRP (ab16284, Abcam). Chemiluminescent signal was developed in Chemidoc Imaging system (Biorad) using SuperSignal™ West Pico PLUS Chemiluminescent Substrate (34579, ThermoFisher Scientific) or SuperSignal™ West Femto Maximum Sensitivity Substrate (34094, ThermoFisher Scientific). For western blot quantification, densitometry was performed on raw data images using FIJI software. Uncropped blots from all experimental repeats are provided in the Source Data file.

### Statistics and reproducibility

All statistical analyses were performed using the GraphPad Prism software (version 9). Data following normal distribution were analysed with Student’s *t* test, one-way ANOVA or two-way ANOVA, followed by multiple comparisons test with appropriate post-hoc correction. Data not following normal distribution were analysed with the Mann-Whitney test or Kruskal-Wallis test with Dunn’s multiple comparisons test. Qualitative data were analysed with chi-square test. Survival curves of lifespan and pharmacological paralyses assays were analysed by the Log-rank (Mantel-Cox) test.

Microscopy images are representative of multiple worms assessed across at least two independent experiments.

### Reporting summary

Further information on research design is available in the Nature Portfolio Reporting Summary linked to this article.

## Supplementary information

Supplementary Information

Description of Additional Supplementary Files

Supplementary Data 1

Reporting Summary

Transparent Peer Review file

## Source data

Source Data

## Data Availability

All data supporting the findings of this study are available within the paper and its Supplementary Information. Source data are provided as a Source Data file. Source data are provided in this paper.
