# Supplementary material for: CYLD-mediated lysine63 deubiquitination regulates synaptic transmission and autophagy to mitigate age-related sequelae
Source: Nat Commun. 2026 Jun 4;17:7164. doi: 10.1038/s41467-026-73966-5 (PMC13396389; doi:10.1038/s41467-026-73966-5)
Supplement: Supplementary file 1 — Supplementary Information [file 41467_2026_73966_MOESM1_ESM.pdf]

Supplementary Information

CYLD-mediated lysine63 deubiquitination regulates synaptic transmission and autophagy to mitigate age-related sequelae

Sotiriou A.[#], Konstantinidis G.[#] & Tavernarakis N.

Supplementary Figures 1-3

Supplementary Tables 1-3

Supplementary Figures and Legends

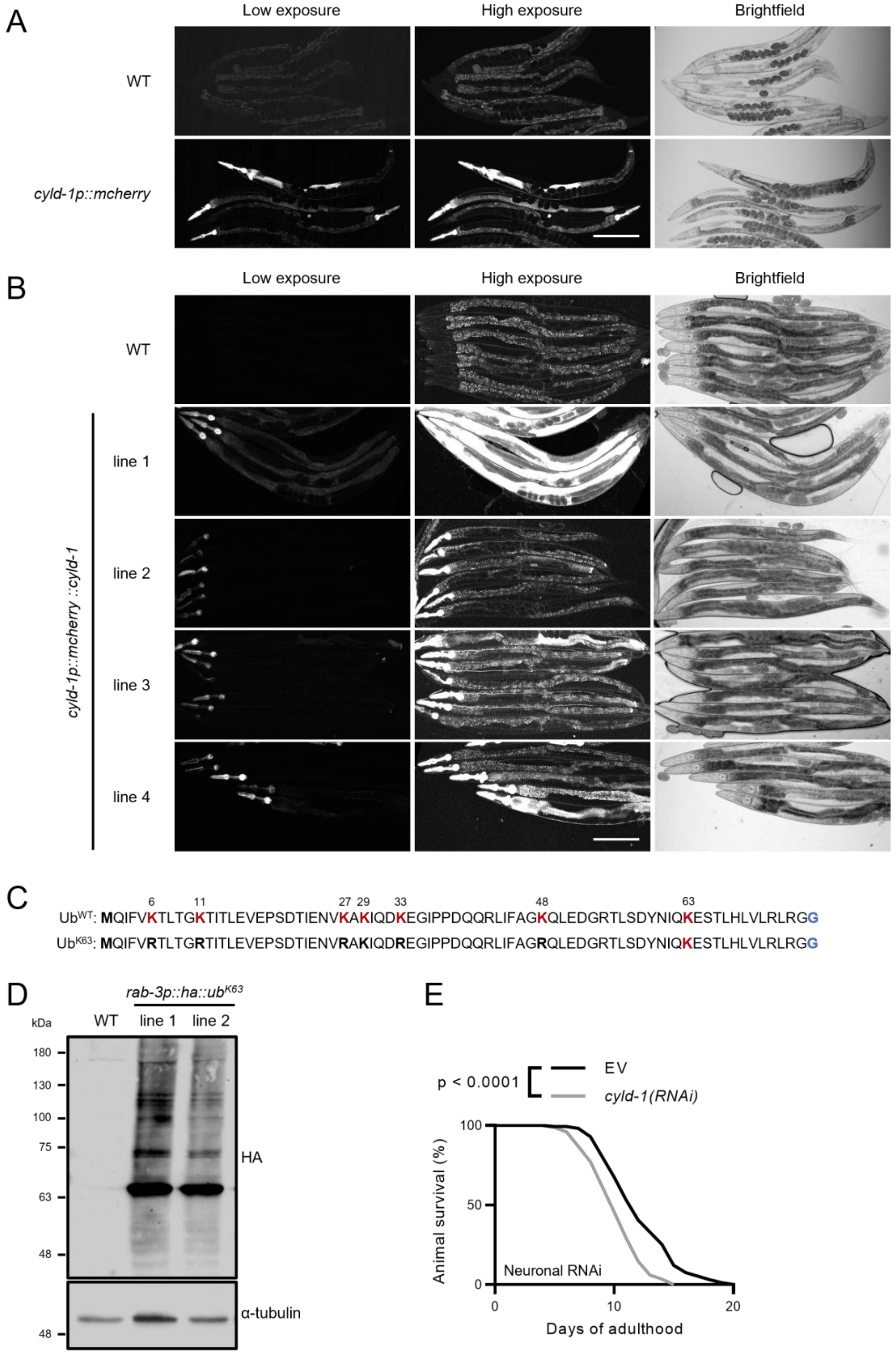

Supplementary Fig. 1: CYLD-1 operates as neuronal K63-specific DUB to promote longevity

A. Low magnification confocal microscopy images of D1 WT and *cyld-1p::mcherry* expressing worms. mCherry fluorescence driven by the *cyld-1* promoter reveals expression in the pharynx, intestine and VNC. Low and high exposure settings are shown. Non-transgenic (WT) animals were used as control for intestinal autofluorescence. Scale bar 200μm.

B. Low magnification confocal microscopy images of D1 WT and *cyld-1p::mcherry::cyld-1* expressing worms. mCherry::CYLD fluorescence driven by the *cyld-1* promoter reveals expression in the pharynx, intestine and VNC. Non-transgenic (WT) animals were used as control for intestinal autofluorescence. Scale bar 200μm.

C. Amino acid sequence of WT and K63 mutant ubiquitin (Ub^{K63}). WT ubiquitin can form polyubiquitin chains of distinct conformations by conjugating the C-terminal glycine (G, shown in blue) of one ubiquitin molecule to any of the seven internal lysines (K6, K11, K27, K29, K33, K48, K63, shown in red) or the N-terminal M1. Mutant ubiquitin (Ub^{K63}) has all internal lysines substituted to arginines, except for K63, therefore it can form only K63-linked polyubiquitin chains.

D. Immunoblot analysis of lysates from WT and *rab-3p::ha::ub*^{K63} worms, detecting HA-tagged neuronal K63-linked polyubiquitin chains and α-tubulin.

E. Survival curves of *sid-1(pk3321); unc-119p::sid-1; myo-2p::mCherry* worms (strain TU3401) treated with control (empty vector) or *cyld-1(RNAi)* from hatching; log rank, Mantel-Cox test.

Source data are provided as a Source Data file.

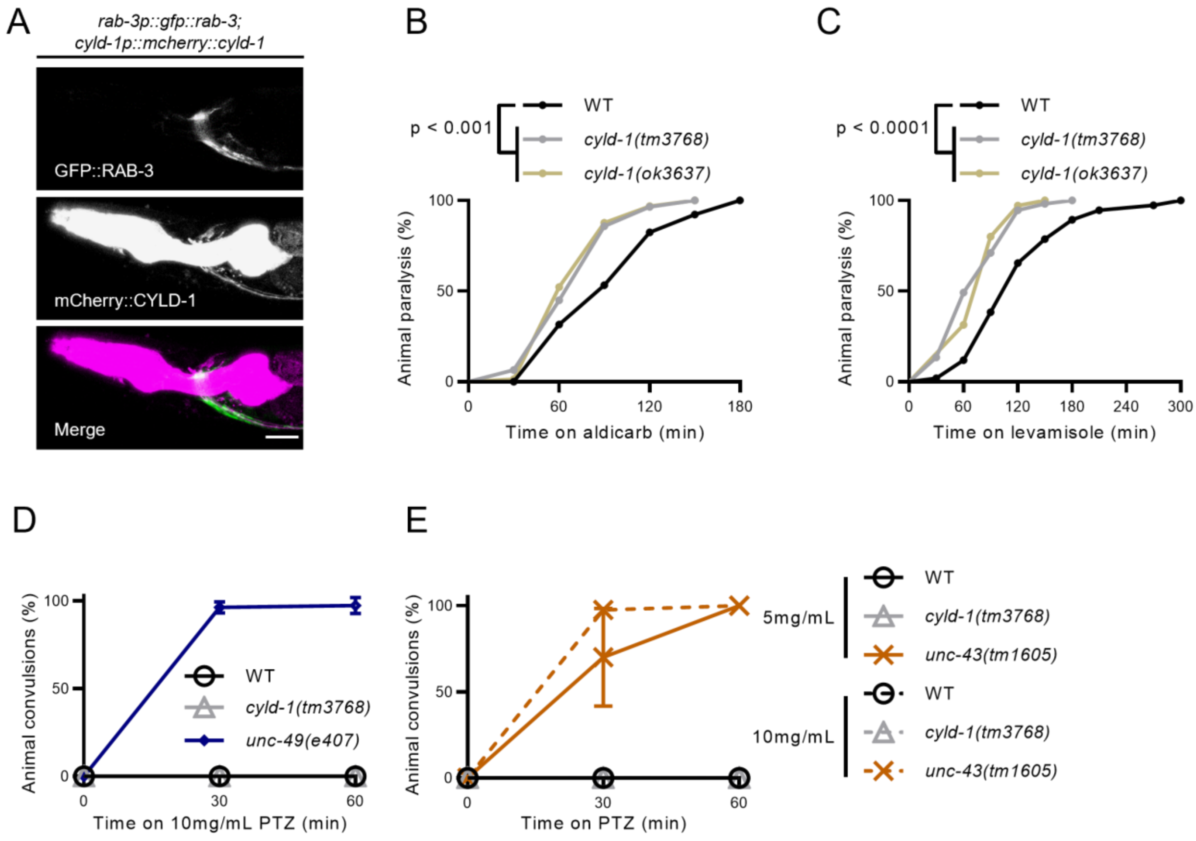

Supplementary Fig. 2: CYLD-1 modulates synaptic function

A. Confocal microscopy images (maximum projection) of the head area of D1 *rab-3p::gfp::rab-3; cyld-1p::mcherry::cyld-1* worms, showing the GFP-tagged synaptic vesicle protein RAB-3 expressed under the control of its endogenous promoter localized in the nerve ring and mCherry-tagged CYLD-1 expressed under the control of its endogenous promoter localized in the nerve ring and pharynx. Scale bar, 10µm.

B. Time-course paralysis assay on 1mM aldicarb of D1 WT, *cyld-1(tm3768)* and *cyld-1(ok3637)* worms; log rank, Mantel-Cox test.

C. Time-course paralysis assay on 0.1mM levamisole of D1 WT, *cyld-1(tm3768)* and *cyld-1(ok3637)* worms; log rank, Mantel-Cox test.

D. Quantification of the epileptic-like behaviour on 10mg/mL PTZ on plates of D1 WT, *cyld-1(tm3768)* and *unc-49(e407)* worms.

E. Quantification of the epileptic-like behaviour on 5mg/mL and 10mg/mL PTZ in liquid of D1 WT, *cyld-1(tm3768)* and *unc-43(tm1605)* worms.

Data presented as mean ± SD. Source data are provided as a Source Data file.

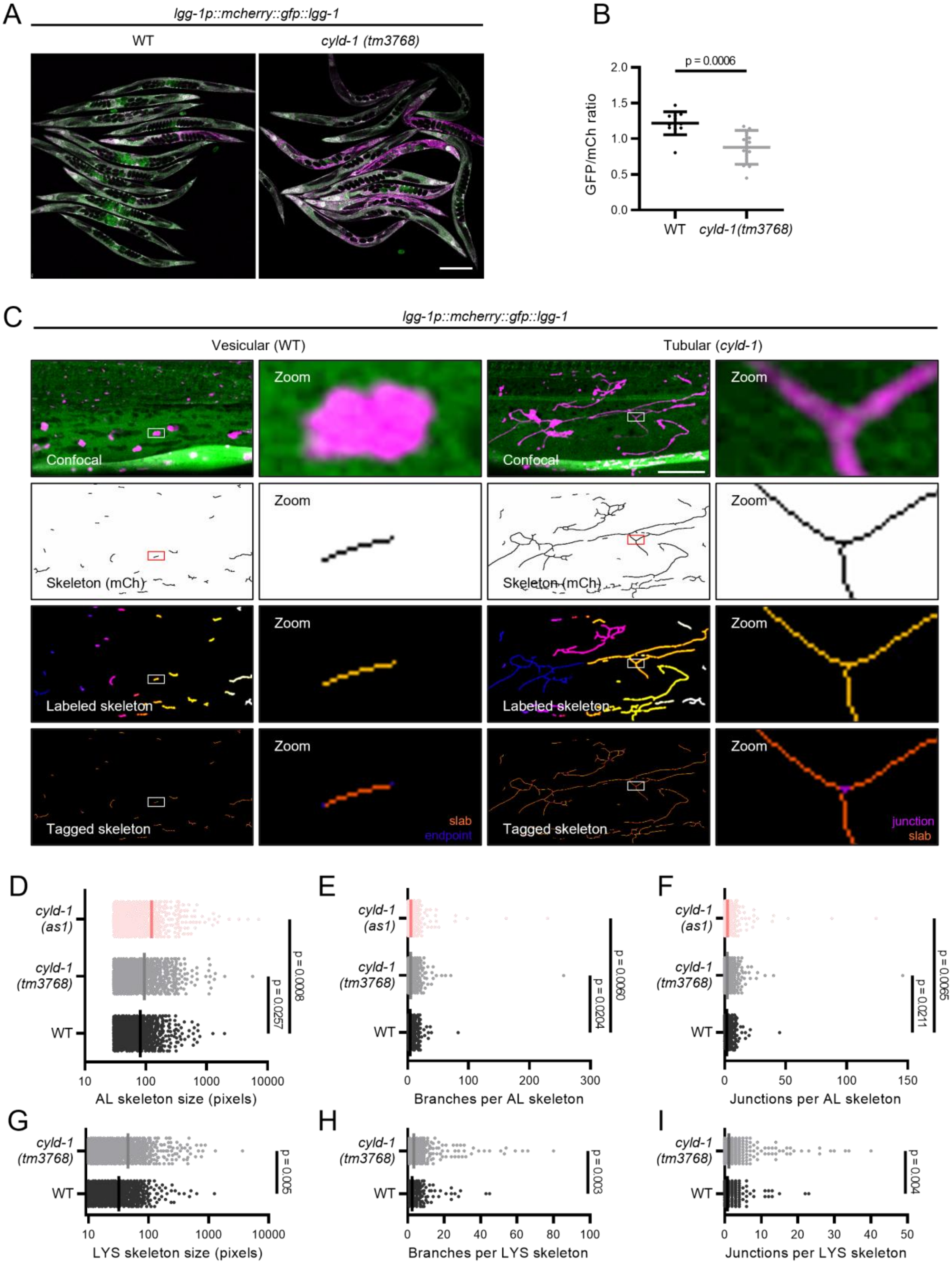

Sotiriou#, Konstantinidis# & Tavernarakis

Supplementary Fig. 3: Morphometric analysis of autolysosomes upon CYLD-1 deficiency

A. Confocal microscopy images of D1 WT and *cyld-1(tm3768)* worms expressing *mcherry::gfp::lgg-1* under the control of the *lgg-1* promoter, showing the mCherry-GFP-tagged autophagosomal protein LGG-1. mCherry only fluorescence dominance indicates autolysosome enrichment. Scale bar, 200μm.

B. Quantification of the ratio of GFP to mCherry mean pixel intensity from (**A**) (n≥11 worms; Two-sided Student's *t*-test with Welch's correction).

C. Skeleton morphometric analysis of confocal microscopy images related to Fig. 6G. In the confocal microscopy image (first row) autolysosomes appear as mCherry only positive structures. Autolysosomal structures were skeletonized (second row). Each skeleton was differentially colour labelled (third row). For every skeleton (fourth row), each pixel was classified as endpoint (blue), slab (orange) or junction (purple). Scale bar, 10μm.

D. Quantification of the autolysosomal skeleton size from Fig. 6G (n≥818 skeletons; Brown-Forsythe and Welch ANOVA tests).

E. Quantification of the number of branches per autolysosomal skeleton from Fig. 6G (n≥818 skeletons; Brown-Forsythe and Welch ANOVA tests).

F. Quantification of the number of junctions per autolysosomal skeleton from Fig. 6G (n≥818 skeletons; Brown-Forsythe and Welch ANOVA tests).

G. Quantification of the lysosomal skeleton size from Fig. 7A, B (n≥1076 skeletons; Two-sided Mann-Whitney test).

H. Quantification of the number of branches per lysosomal skeleton from Fig. 7A, B (n≥1076 lysosomal skeletons; Two-sided Mann-Whitney test).

I. Quantification of the number of junctions per lysosomal skeleton from Fig. 7A, B (n≥1076 lysosomal skeletons; Two-sided Mann-Whitney test).

Data presented as mean ± SD. Source data are provided as a Source Data file.

Supplementary Tables

Supplementary Table 1: List of strains

Strain name	Genotype	Originator	Figure	Comments
N2	*C. elegans* wild isolate	CGC	1B, 1C, 1D, 1E, 1F, 1G, 1H, 1I, 3G, 4C, 4J, 5G, 5H, 5I, 5J, 5K 5L, 7F, 7G, S1A, S1B, S1D, S2B, S2C, S2D, S2E,	Used as WT in this study
CB4037	*glp-1(e2141ts) III*	CGC	1G	
TU3401	*sid-1(pk3321) V; uIs69[unc-119p::sid-1 + myo-2p::mcherry] V*	CGC	4D, 4E, 4K, S1E	Neuronal RNAi-competent strain
DA2123	*N2; adIs[lgg-1p::gfp::lgg-1 + rol-6]*	CGC	5C, 5D	
DLM1	*unc-119(ed3) III; uwaEx1[eft-3p::cerulean-venus::lgg-1 + unc-119(+)]*	CGC	5E, 5F	
RB2610	*cyld-1(ok3637) III*	CGC	4J, S2B, S2C	
CZ631	*juIs14[acr-2p::gfp + lin-15(+)] IV; lin-15 X*	CGC	3A, 3B, 3E	
RB1206	*rsks-1(ok1255) III*	CGC	5K, 5L	
MAH215	*N2; sqIs11[lgg-1p::mcherry::gfp::lgg-1 + rol-6]*	CGC	6A, 6B, 6C, 6G, 6H, 6I, 6J, 7J, S3A, S3B, S3C, S3D, S3E, S3F	
CB1370	*daf-2(e1370) III*	CGC	1D	
KG2430	*ceIs56[unc-129p::ctns-1::mcherry + unc-129p::nlp-21::venus + ttx-3p::rfp]*	CGC	7H, 7I	
XW5399	*unc-76(e911) V; qxIs257 [ced-1p::nuc-1::mcherry + unc-76(+)]*	CGC	7A, 7B, S3G, S3H, S3I	
MAH508	*N2; sqEx67[rgef-1p::mcherry::gfp::lgg-1 + rol-6]*	CGC	6D, 6E, 6F	
CB407	*unc-49(e407) III*	CGC	S2D	
PHX5270	*ctns-1(syb5270[ctns-1::wrmScarlet]) II*	CGC	7C, 7D, 7E	
TM5755	*lgg-2(tm5755) IV*	Shigen	5H, 5I, 5J	
TM1605	*unc-43(tm1605) IV*	Shigen	S2E	
DA465	*eat-2(ad465) II*	Avery L	1F	
MAH677	*sid-1(qt9) V; sqIs71[rgef-1p::gfp::unc-54 3'UTR + rgef-1p::sid-1::unc-54 3'UTR + pBS]*	Hansen M.	2E	Neuronal RNAi-competent strain
EG8244	*oxSi834[unc-47p::gfp::snb-1 + unc-119(+)] II*	Jorgensen E.	4F, 4G, 4H	

IR2375	*N2; oxIs608[unc-47p::mcherry]*	Nektarios Tavernarakis lab	3C, 3D, 3F	Obtained from the strain EG6531: *N2; oxIs608[unc-47p::mCherry]; oxEx1182[unc-47p::TOMM-20::GFP]*
IR2379	*N2; Ex[rab-3p::dsRed::lgg-1]*	Nektarios Tavernarakis lab	5A, 5B	
IR2846	*cyld-1(tm3768); adIs[lgg-1p::gfp::lgg-1 + rol-6]*	This study	5C, 5D	DA2123 x IR2847
IR2847	*cyld-1(tm3768) III outcrossed x3*	This study	1B, 1C, 1E, 1H, 1I, 3G, 4C, 4J, 5G, 5H, 5I, 5J, 5K 5L, 7F, 7G, S2B, S2C, S2D, S2E	TM3768 x N2 (three times)
IR2952	*unc-119(ed3) III; Ex[cyld-1p::mcherry + unc-119 (+)]*	This study	2A, S1A	Generated by bombardment of plasmids #2899 + pPK719
IR2960	*juIs14[acr-2p::gfp + lin-15(+)] IV; Ex1[cyld-1p::mcherry + unc-119 (+)]*	This study	2B	IR2952 x CZ631
IR2963	*cyld-1(tm3768) III; juIs14[acr-2p::gfp + lin-15(+)] IV*	This study	3A, 3B, 3E	IR2847 x CZ631
IR2964	*cyld-1(tm3768) III; oxIs608[unc-47p::mcherry]*	This study	3C, 3D, 3F	IR2847 x IR2375
IR3014	*oxSi834[unc-47p::gfp::snb-1 + unc-119(+)] II; cyld-1(tm3768) III*	This study	4F, 4G, 4H	EG8244 x IR2847
IR3052	*unc-119(ed3) III; Ex[cyld-1p::mcherry::cyld-1 + unc-119(+)] line 1*	This study	S1B	Generated by bombardment of plasmids #3102 + pPK719
IR3053	*unc-119(ed3) III; Ex[cyld-1p::mcherry::cyld-1 + unc-119(+)] line 2*	This study	1C, S1B	Generated by bombardment of plasmids #3102 + pPK719
IR3054	*unc-119(ed3) III; Ex[cyld-1p::mcherry::cyld-1 + unc-119(+)] line 3*	This study	S1B	Generated by bombardment of plasmids #3102 + pPK719
IR3055	*unc-119(ed3) III; Ex[cyld-1p::mcherry::cyld-1 + unc-119(+)] line 4*	This study	S1B	Generated by bombardment of plasmids #3102 + pPK719
IR3056	*cyld-1(tm3768) III; lgg-2(tm5755) IV*	This study	5H, 5I, 5J	TM5755 x IR2847
IR3060	*unc-119(ed3) III; Ex[rab-3p::ha::ub^{K63} + unc-119(+)] line 1*	This study	S1D	Generated by bombardment of plasmids #3114 + pPK719

IR3062	*unc-119(ed3) III; Ex[rab-3p::ha::ub^{K63} + unc-119(+)] line 2*	This study	S1D	Generated by bombardment of plasmids #3114 + pPK719
IR3069	*cyld-1(tm3768) III; sqIs11[lgg-1p::mcherry::gfp::lgg-1 + rol-6]*	This study	6A, 6B, 6C, 6G, 6H, 6I, 6J, S3A, S3B, S3C, S3D, S3E, S3F	MAH215 x IR2847
IR3071	*cyld-1(tm3768) III; sqEx67[rgef-1p::mcherry::gfp::lgg-1 + rol-6]*	This study	6D, 6E, 6F	MAH508 x IR2847
IR3072	*hpIs3[unc-25p::syd-2::gfp] X; Ex[cyld-1p::mcherry::cyld-1 + unc-119(+)]*	This study	4B	IR3053 x ZM54
IR3073	*jsIs682[rab-3p::gfp::rab-3 + lin15(+)] III; Ex[cyld-1p::mcherry::cyld-1 + unc-119(+)]*	This study	4A, S2A	IR3053 x NM2415
IR3150	*cyld-1(tm3768) III; Ex[rab-3p::dsRed::lgg-1]*	This study	5A, 5B	IR2379 x IR2847
IR3157	*unc-119(ed3) III; Is[rab-3p::ha::ub^{K63} + myo-2p::gfp + unc-119(+)]*	This study	2C, 2D	Generated by bombardment of plasmids #3114 + pPD112.11 + pPK719. Random integration
IR3161	*cyld-1(tm3768) III; Is[rab-3p::ha::ub^{K63} + myo-2p::gfp + unc-119(+)]*	This study	2C, 2D	IR3157 x IR2847
IR3164	*cyld-1(tm3768) III; qxIs257 [ced-1p::nuc-1::mcherry + unc-76(+)]*	This study	7A, 7B, S3G, S3H, S3I	XW5399 x IR2847
IR3173	*cyld-1(tm3768) III; Ex[cyld-1p::mcherry::cyld-1 + unc-119(+)]*	This study	1C	IR3053 x IR2847
IR3254	*cyld-1(as1[cyld-1[C774S]) III line 1*	This study	1B, 1H, 1I, 4C, 5K, 5L, 7F, 7G	Generated by CRISPR-Cas9 mutagenesis to modify endogenous *cyld-1* locus, outcrossed three times to N2
IR3255	*cyld-1(as1[cyld-1[C774S]) III line 2*	This study	1B, 4C, 5K, 5L	Generated by CRISPR-Cas9 mutagenesis to modify endogenous *cyld-1* locus, outcrossed two times to N2
IR3264	*cyld-1(tm3768); ceIs56[unc-129p::ctns-1::mcherry + unc-129p::nlp-21::venus + ttx-3p::rfp]*	This study	7H, 7I	KG2430 x IR2847

IR3293	*cyld-1(as1[C774S]) III; sqIs11[lgg-1p::mcherry::gfp::lgg-1 + rol-6]*	This study	6G, 6H, 6I, 6J, S3D, S3E, S3F	MAH215 x IR3254
IR3367	*ctns-1(syb5270[ctns-1::wrmScarlet]) II; cyld-1(tm3768) III*	This study	7C, 7D, 7E	PHX5270 x IR2847
IR3371	*cyld-1(ok3637) III outcrossed x2*	This study	1B, 5K, 5L	RB2610 x N2 (two times)
IR3372	*cyld-1(ok3637) III; oxIs608[unc-47p::mcherry]*	This study	3C, 3D, 3F	IR2375 x IR3371
IR3373	*cyld1(ok3637) III; juIs14[acr-2p::gfp + lin-15(+)] IV; lin-15 X*	This study	3A, 3B, 3C	CZ631 x IR3371
IR3398	*sid-1(qt9) V; sqIs71[rgef-1p::gfp::unc-54 3'UTR + rgef-1p::sid-1::unc-54 3'UTR + pBS]; sqIs11[lgg-1p::mcherry::gfp::lgg-1 + rol-6]*	This study	7J	MAH215 x MAH677

Supplementary Table 2: List of oligonucleotides

Oligo name	Sequence
	Genotyping
tm3768 FW	GATATTGAGAATCTTTTGCGTG
tm3768 RV	GATTTTCTGATTGATTCGTATGA
tm3768int FW	TCATTTTGCATTGAATAATCTG
tm3768int RV	CTTTCGATATAAATAAGAATAAATTG
tm5755 FW	GGAAATCGGGGAGGATCTTACA
tm5755 RV	CAATGTTCAGGGCAGTAAGGA
ok3637 FW	GAAGCACAATCATTCGGGATC
ok3637 RV	AAACGATTCCATCCTTGCAG
	Cloning
cyld-1p FW	ACCGGTTCTGAAATATGCGGGTTGTTTAG
cyld-1p RV	TCTAGAGGTCTTTCCGTTTCCTCGAC
cyld-1 full cds FW	GCTAGCATGCCCGGCGAATTTGC
cyld-1 full cds RV	GCTAGCCTAGTTCATCATATTTTTAATCGAGC
HA KpnI FW	CCCGGTACCATGGGCTACCCCTATGA
Ubi NheI RV	AATAGCTAGCTCAACCACCTCTGAGACG
	RNAs
tracrRNA	IDT # 1072532
cyld-1C774S crRNA	GACAAACATTGCATACAACG
dpy-10 crRNA	GCTACCATAGGCACCACGAG
	ssODNs
cyld-1C774S ssODN	AGTATGGATTCAGGAGTGGAGAAGCAAAAATGTGGAATTGCAAAGGACATG CAACAATTAGTTGGCAGGCAGAAAGGCATTCAGGGTTACTGTAACTCGTCTT ATCTAGATGCGACCTTGTATGCAATGTTTGTGCAAACCACTTGCTTTGATTTG TAAGTTTGGCATGCTCTCAATAAAAAATTTTAAAATATAATTTC
dpy-10 ssODN	CACTTGAACTTCAATACGGCAAGATGAGAATGACTGGAAACCGTACCGCATG CGGTGCCTATGGTAGCGGAGCTTCACATGGCTTCAGACCAACAGCCTAT

Supplementary Table 3: Lifespan statistics

Figure	Experiment-Repeat	Strain	Genotype /Treatment	Median lifespan	P value	Deaths	Censored	Total
1B	1.1	N2	WT	15		94	28	122
		IR2847	*cyld-1(tm3768)*	9	vs WT <0.0001	67	37	104
		IR3371	*cyld-1(ok3637)*	11	vs WT <0.0001	54	30	84
		IR3254	*cyld-1(as1) line 1*	11	vs WT <0.0001	83	26	109
		IR3255	*cyld-1(as1) line 2*	11	vs WT <0.0001	61	17	78
	1.2	N2	WT	15		88	32	120
		IR2847	*cyld-1(tm3768)*	10	vs WT <0.0001	49	35	84
		IR3371	*cyld-1(ok3637)*	10	vs WT <0.0001	46	36	82
		IR3254	*cyld-1(as1) line 1*	9	vs WT <0.0001	69	36	105
		IR3255	*cyld-1(as1) line 2*	9	vs WT <0.0001	38	28	66
1C	2.1	N2	WT	14		74	44	118
		IR2847	*cyld-1(tm3768)*	10	vs WT <0.0001	66	27	93
		IR3053	*cyld-1p::mcherry::cyld-1*	14		57	59	116
		IR3173	*cyld-1(tm3768); cyld-1p::mcherry::cyld-1*	12	vs *cyld-1(tm3768)* <0.0001	81	58	139
	2.2	N2	WT	11		109	22	131
		IR2847	*cyld-1(tm3768)*	9	vs WT <0.0001	111	12	123
		IR3053	*cyld-1p::mcherry::cyld-1*	11		62	16	78
		IR3173	*cyld-1(tm3768); cyld-1p::mcherry::cyld-1*	11	vs *cyld-1(tm3768)* <0.0001	46	18	64
1D	3.1	N2	WT; EV	16		134	26	160
		N2	WT; *cyld-1(RNAi)*	14	vs WT; EV <0.0001	93	34	127
		CB1370	*daf-2(e1370);* EV	31		59	16	75

		CB1370	*daf-2(e1370); cyld-1(RNAi)*	20	vs *daf-2 (e1370)*; EV <0.0001<br><br>vs WT; *cyld-1(RNAi)* <0.0001	63	15	78
	3.2	N2	WT; EV	15		153	26	179
		N2	WT; *cyld-1(RNAi)*	13	vs WT; EV <0.0001	109	17	126
	3.3	CB1370	*daf-2(e1370);* EV	40		51	20	71
		CB1370	*daf-2(e1370); cyld-1(RNAi)*	24	vs *daf-2 (e1370)*; EV <0.0001	45	17	62
	3.4	N2	WT; EV	12		39	24	63
		N2	WT; *cyld-1(RNAi)*	10	vs WT; EV < 0.0001	70	5	75
1E	4.1	N2	WT; EV (*same as in exp 3.1)	16		134	26	160
		N2	WT; *daf-2(RNAi)*	27	vs WT; EV <0.0001	88	51	139
		IR2847	*cyld-1(tm3768);* EV	12		84	14	98
		CB1370	*cyld-1(tm3768); daf-2(RNAi)*	20	vs *daf-2 (e1370)*; EV <0.0001<br><br>vs WT; *daf-2(RNAi)* <0.0001	80	15	95
1F	5.1	N2	WT; EV	14		97	6	103
		N2	WT; *cyld-1(RNAi)*	12	vs WT; EV <0.0001	92	9	101
		DA465	*eat-2(ad465);* EV	18		61	31	92
		DA465	*eat-2(ad465); cyld-1(RNAi)*	14	vs *eat-2 (ad465)*; EV <0.0001<br><br>vs WT; *cyld-1(RNAi)* <0.0001	59	43	102
	5.2	N2	WT; EV	14		71	5	76
		N2	WT; *cyld-1(RNAi)*	12	vs WT; EV <0.0001	66	10	76
		DA465	*eat-2(ad465);* EV	18		84	70	154
		DA465	*eat-2(ad465); cyld-1(RNAi)*	14	vs *eat-2 (ad465)*; EV <0.0001	86	60	146

					vs WT; *cyld-1(RNAi)* <0.0001			
1G	6.1	N2	WT; EV	17		75	25	100
		N2	WT; *cyld-1(RNAi)*	9	vs WT; EV <0.0001	79	17	96
		CB4037	*glp-1(e2141ts);* EV	21		104	15	119
		CB4037	*glp-1(e2141ts); cyld-1(RNAi)*	11	vs *glp-1(e2141ts)*; EV <0.0001<br><br>vs WT; *cyld-1(RNAi)* =0.0087	118	1	119
	6.2	N2	WT; EV (*same as in exp 5.1)	14		71	5	76
		N2	WT; *cyld-1(RNAi)* (*same as in exp 5.1)	12	vs WT; EV <0.0001	66	10	76
		CB4037	*glp-1(e2141ts);* EV	19		113	10	123
		CB4037	*glp-1(e2141ts); cyld-1(RNAi)*	12	vs *glp-1(e2141ts)*; EV <0.0001<br><br>vs WT; *cyld-1(RNAi)* =0.0042	106	3	109
2E	7.1	MAH667	EV	18		140	6	146
		MAH667	*cyld-1(RNAi)*	15	vs EV <0.0001	107	7	114
	7.2	MAH667	EV	18		120	22	142
		MAH667	*cyld-1(RNAi)*	16	vs EV 0.0003	115	9	124
5G	8.1	N2	WT; EV	18		95	15	110
		N2	WT; *unc-51(RNAi)*	13	vs WT; EV 0.0003	117	2	119
		IR2847	*cyld-1(tm3768);* EV	9		68	35	103
		IR2847	*cyld-1(tm3768); unc-51(RNAi)*	9	*vs cyld-1(tm3768);* EV 0.4968	69	30	99
	8.2	N2	WT; EV	16		102	7	109
		N2	WT; *unc-51(RNAi)*	14	vs EV <0.0001	120	4	124
		IR2847	*cyld-1(tm3768);* EV	11		75	5	80
		IR2847	*cyld-1(tm3768); unc-51(RNAi)*	11	vs *cyld-1(tm3768)*; EV 0.11	72	2	74

5H	9.1	N2	WT	11		137	46	183
		TM5755	*lgg-2(tm5755)*	9	vs WT <0.0001	66	14	80
		IR2847	*cyld-1(tm3768)*	9		82	46	128
		IR3056	*cyld-1(tm3768); lgg-2(tm5755)*	8	vs *cyld-1(tm3768)* 0.5461	39	66	105
	9.2	N2	WT (*same as in experiment 1.1)	11		109	22	131
		TM5755	*lgg-2(tm5755)*	9	vs WT <0.0001	85	8	93
	9.3	IR2847	*cyld-1(tm3768)*	10		42	43	85
		IR3056	*cyld-1(tm3768); lgg-2(tm5755)*	10	vs *cyld-1(tm3768)* 0.3329	26	55	81
S1E	10.1	TU3401	EV	12		161	22	183
		TU3401	*cyld-1(RNAi)*	10	vs EV <0.0001	126	54	180
	10.2	TU3401	EV	14		59	26	85
		TU3401	*cyld-1(RNAi)*	10	vs EV <0.0001	63	19	82
