# Supplementary material for: CYLD-mediated lysine63 deubiquitination regulates synaptic transmission and autophagy to mitigate age-related sequelae
Source: Nat Commun. 2026 Jun 4;17:7164. doi: 10.1038/s41467-026-73966-5 (PMC13396389; doi:10.1038/s41467-026-73966-5)
Supplement: Supplementary file 2 — Description of Additional Supplementary Files [file 41467_2026_73966_MOESM2_ESM.pdf]

## Description of Additional Supplementary Files

**File name: Supplementary Data 1**
Description: Detailed assessment of cholinergic and GABAergic motor neuron degeneration in young and old worms.
