# Supplementary material for: CYLD-mediated lysine63 deubiquitination regulates synaptic transmission and autophagy to mitigate age-related sequelae
Source: Nat Commun. 2026 Jun 4;17:7164. doi: 10.1038/s41467-026-73966-5 (PMC13396389; doi:10.1038/s41467-026-73966-5)
Supplement: Supplementary file 4 — Reporting Summary [file 41467_2026_73966_MOESM4_ESM.pdf]

nature portfolio

Corresponding author(s): Nektarios Tavernarakis

Last updated by author(s): Apr 30, 2026

Reporting Summary

Nature Portfolio wishes to improve the reproducibility of the work that we publish. This form provides structure for consistency and transparency in reporting. For further information on Nature Portfolio policies, see our Editorial Policies and the Editorial Policy Checklist.

Statistics

For all statistical analyses, confirm that the following items are present in the figure legend, table legend, main text, or Methods section.

n/a	Confirmed	
☐	☒	The exact sample size (*n*) for each experimental group/condition, given as a discrete number and unit of measurement
☐	☒	A statement on whether measurements were taken from distinct samples or whether the same sample was measured repeatedly
☐	☒	The statistical test(s) used AND whether they are one- or two-sided<br>*Only common tests should be described solely by name; describe more complex techniques in the Methods section.*
☒	☐	A description of all covariates tested
☐	☒	A description of any assumptions or corrections, such as tests of normality and adjustment for multiple comparisons
☐	☒	A full description of the statistical parameters including central tendency (e.g. means) or other basic estimates (e.g. regression coefficient) AND variation (e.g. standard deviation) or associated estimates of uncertainty (e.g. confidence intervals)
☐	☒	For null hypothesis testing, the test statistic (e.g. *F*, *t*, *r*) with confidence intervals, effect sizes, degrees of freedom and *P* value noted<br>*Give P values as exact values whenever suitable.*
☒	☐	For Bayesian analysis, information on the choice of priors and Markov chain Monte Carlo settings
☒	☐	For hierarchical and complex designs, identification of the appropriate level for tests and full reporting of outcomes
☒	☐	Estimates of effect sizes (e.g. Cohen's *d*, Pearson's *r*), indicating how they were calculated

Our web collection on statistics for biologists contains articles on many of the points above.

Software and code

Policy information about availability of computer code

Data collection	ZEN 3.5 (blue edition) Version 3.5.93.00009 (AxioImager Z2)<br>ZEN 3.6 (blue edition) Version 3.6.095.09000 (LSM900)<br>Leica Application Suite X Version 3.5.723225 (LEICA SP8)<br>Image LabTM Touch Software Version 2.0.0.27 (ChemiDoc Imaging System)<br>DiamondScope 2.0.883.0 (EVOS Invitrogen FL Auto 2.0 Imaging System)
Data analysis	FIJI 1.54f - Java 1.8.0_322 64 bit (image processing/analysis)<br>Benchling [Biology Software] (2025), retrieved from https://benchling.com (cloning and CRISPR design)<br>GraphPad Prism 9.0 (statistical analysis)

For manuscripts utilizing custom algorithms or software that are central to the research but not yet described in published literature, software must be made available to editors and reviewers. We strongly encourage code deposition in a community repository (e.g. GitHub). See the Nature Portfolio guidelines for submitting code & software for further information.

Data

Policy information about availability of data

All manuscripts must include a data availability statement. This statement should provide the following information, where applicable:

- Accession codes, unique identifiers, or web links for publicly available datasets
- A description of any restrictions on data availability
- For clinical datasets or third party data, please ensure that the statement adheres to our policy

The authors declare that all data supporting the findings of this study are available within the paper and its supplementary information files.

Research involving human participants, their data, or biological material

Policy information about studies with human participants or human data. See also policy information about sex, gender (identity/presentation), and sexual orientation and race, ethnicity and racism.

Reporting on sex and gender	No human participants were used in this study.
Reporting on race, ethnicity, or other socially relevant groupings	No human participants were used in this study.
Population characteristics	No human participants were used in this study.
Recruitment	No human participants were used in this study.
Ethics oversight	No human participants were used in this study.

Note that full information on the approval of the study protocol must also be provided in the manuscript.

Field-specific reporting

Please select the one below that is the best fit for your research. If you are not sure, read the appropriate sections before making your selection.

☒ Life sciences ☐ Behavioural & social sciences ☐ Ecological, evolutionary & environmental sciences

For a reference copy of the document with all sections, see nature.com/documents/nr-reporting-summary-flat.pdf

Life sciences study design

All studies must disclose on these points even when the disclosure is negative.

Sample size	Exact sample size for each experiment is reported the figure legends or supplementary tables of the manuscript.<br>For lifespan experiments more than 100 animals were used per individual experiment, which exceeds the typical standard in the field.<br>Fluorescence imaging experiments were replicated at least two independent times, with as many animals as could reasonably be analyzed by the analysis methods available.<br>Maximum available samples were used for all experiments.
Data exclusions	No data exclusion was performed in this study.
Replication	Multiple replicates were performed for all experiments as mentioned in methods and figure legends.
Randomization	Several strains used in this study can be identified and distributed to experimental groups based on specific fluorescent reporter expression and/or phenotype.
Blinding	During all experiments with objective measurements (such as microscopy and lifespan assays), the experimenter was blinded regarding the experimental samples.

Reporting for specific materials, systems and methods

We require information from authors about some types of materials, experimental systems and methods used in many studies. Here, indicate whether each material, system or method listed is relevant to your study. If you are not sure if a list item applies to your research, read the appropriate section before selecting a response.

Materials & experimental systems

n/a	Involved in the study
☐	☒ Antibodies
☒	☐ Eukaryotic cell lines
☒	☐ Palaeontology and archaeology
☐	☒ Animals and other organisms
☒	☐ Clinical data
☒	☐ Dual use research of concern
☒	☐ Plants

Methods

n/a	Involved in the study
☒	☐ ChIP-seq
☒	☐ Flow cytometry
☒	☐ MRI-based neuroimaging

Antibodies

Antibodies used

Western blot – Primary antibodies:
Rabbit anti-GFP, Minotech, #701, https://minotech.gr/index.php/products/anti-hrcn, 1:10000.
Rabbit anti-HA-Tag (C29F4), Cell Signaling, #3724, RRID: AB_1549585, 1:1000.
Rabbit anti-Ubiquitin Lys63-Specific (Apu3), Merck, #05-1308, AB_1587580, 1:1000.
Mouse anti-α-Tubulin, DSHB, #12G10, RRID: AB_1157911, 1:10000.
Rabbit anti-phospho-p70 S6 Kinase (Ser371), Cell Signaling, #9208, RRID:AB_330990, 1:1000)
Western blot – Secondary antibodies:
Goat anti-Mouse HRP, Abcam, #ab6789, RRID: AB_955439, 1:10000.
Donkey anti-Rabbit HRP, Abcam, #ab16284, RRID: AB_955387, 1:10000.

Validation

All the antibodies used in this study are commercially available and validation were performed by the manufacturers and supported by the publications indicated in the manufacturers' websites.

Animals and other research organisms

Policy information about studies involving animals; ARRIVE guidelines recommended for reporting animal research, and Sex and Gender in Research

Laboratory animals

Caenorhabditis elegans:
Provided by the Caenorhabditis Genetics Center (CGC):
N2: C. elegans wild isolate
CB4037: glp-1(e2141ts) III
TU3401: sid-1(pk3321) V; uIs69[unc-119p::sid-1 + myo-2p::mcherry] V
DA2123: N2; adIs[lgg-1p::gfp::lgg-1 + rol-6]
DLM1: unc-119(ed3) III; uwaEx1[eft-3p::cerulean-venus::lgg-1 + unc-119(+)]
RB2610: cyld-1(ok3637) III
CZ631: juIs14[acr-2p::gfp + lin-15(+)] IV; lin-15 X
RB1206: rsks-1(ok1255) III
MAH215: N2; sqIs11[lgg-1p::mcherry::gfp::lgg-1 + rol-6]
CB1370: daf-2(e1370) III
KG2430: ceIs56[unc-129p::ctns-1::mcherry + unc-129p::nlp-21::venus + ttx-3p::rfp]
XW5399: unc-76(e911) V; qxIs257 [ced-1p::nuc-1::mcherry + unc-76(+)]
MAH508: N2; sqEx67[rgef-1p::mcherry::gfp::lgg-1 + rol-6]
CB407: unc-49(e407) III
PHX5270: ctns-1(syb5270[ctns-1::wrmScarlet]) II

Provided by S. Mitani (National Bioresource Project) in Japan (Shigen):
TM5755: lgg-2(tm5755) IV
TM1605: unc-43(tm1605) IV

Provided by M. Hansen:
MAH677: sid-1(qt9) V; sqIs71[rgef-1p::gfp::unc-54 3'UTR + rgef-1p::sid-1::unc-54 3'UTR + pBS]

Provided by L. Avery:
DA465: eat-2(ad465) II

Provided by E. Jorgensen:
EG8244: oxSi834[unc-47p::gfp::snb-1 + unc-119(+)] II

Generated in N. Tavernarakis laboratory:
IR2375: N2; oxIs608[unc-47p::mcherry]
IR2379: N2; Ex[rab-3p::dsRed::lgg-1]
IR2846: cyld-1(tm3768); adIs[lgg-1p::gfp::lgg-1 + rol-6]
IR2847: cyld-1(tm3768) III outcrossed x3
IR2952: unc-119(ed3) III; Ex[cyld-1p::mcherry + unc-119 (+)]
IR2960: juIs14[acr-2p::gfp + lin-15(+)] IV; Ex1[cyld-1p::mcherry + unc-119 (+)]

IR2963: cyld-1(tm3768) III; juIs14[acr-2p::gfp + lin-15(+)] IV
IR2964: cyld-1(tm3768) III; oxIs608[unc-47p::mcherry]
IR3014: oxSi834[unc-47p::gfp::snb-1 + unc-119(+)] II; cyld-1(tm3768) III
IR3052: unc-119(ed3) III; Ex[cyld-1p::mcherry::cyld-1 + unc-119(+)]
IR3053: unc-119(ed3) III; Ex[cyld-1p::mcherry::cyld-1 + unc-119(+)]
IR3054: unc-119(ed3) III; Ex[cyld-1p::mcherry::cyld-1 + unc-119(+)]
IR3055: unc-119(ed3) III; Ex[cyld-1p::mcherry::cyld-1 + unc-119(+)]
IR3056: cyld-1(tm3768) III; lgg-2(tm5755) IV
IR3060: unc-119(ed3) III; Ex[rab-3p::ha::ubK63 + unc-119(+)] line 4
IR3062: unc-119(ed3) III; Ex[rab-3p::ha::ubK63 + unc-119(+)] line 6
IR3069: cyld-1(tm3768) III; sqIs11[lgg-1p::mcherry::gfp::lgg-1 + rol-6]
IR3071: cyld-1(tm3768) III; sqEx67[rgef-1p::mcherry::gfp::lgg-1 + rol-6]
IR3072: hpIs3[unc-25p::syd-2::gfp] X; Ex[cyld-1p::mcherry::cyld-1 + unc-119(+)]
IR3073: jsIs682[rab-3p::gfp::rab-3 + lin15(+)] III; Ex[cyld-1p::mcherry::cyld-1 + unc-119(+)]
IR3150: cyld-1(tm3768) III; Ex[rab-3p::dsRed::lgg-1]
IR3157: unc-119(ed3) III; Is[rab-3p::ha::ubK63 + myo-2p::gfp + unc-119(+)]
IR3161: cyld-1(tm3768) III; Is[rab-3p::ha::ubK63 + myo-2p::gfp + unc-119(+)]
IR3164: cyld-1(tm3768) III; qxIs257 [ced-1p::nuc-1::mcherry + unc-76(+)]
IR3173: cyld-1(tm3768) III; Ex[cyld-1p::mcherry::cyld-1 + unc-119(+)]
IR3254: cyld-1(as1[cyld-1[C774S]) III line 1
IR3255: cyld-1(as1[cyld-1[C774S]) III line 2
IR3264: cyld-1(tm3768); ceIs56[unc-129p::ctns-1::mcherry + unc-129p::nlp-21::venus + ttx-3p::rfp]
IR3293: cyld-1(as1[C774S]) III; sqIs11[lgg-1p::mcherry::gfp::lgg-1 + rol-6]
IR3367: ctns-1(syb5270[ctns-1::wrmScarlet]) II; cyld-1(tm3768) III
IR3371: cyld-1(ok3637) III outcrossed x2
IR3372: cyld-1(ok3637) III; oxIs608[unc-47p::mcherry]
IR3373: cyld1(ok3637) III; juIs14[acr-2p::gfp + lin-15(+)] IV; lin-15 X
IR3398: sid-1(qt9) V; sqIs71[rgef-1p::gfp::unc-54 3'UTR + rgef-1p::sid-1::unc-54 3'UTR + pBS]; sqIs11[lgg-1p::mcherry::gfp::lgg-1 + rol-6]
Caenorhabditis elegans hermaphrodites at day 1 of adulthood grown at 20°C were used unless otherwise specified.

Wild animals

No wild animals were used in this study.

Reporting on sex

Caenorhabditis elegans is hermaphrodite species and hermaphrodite animals were used for experiments. Sex-based analyses were not performed. No sex-specific reporting is included.

Field-collected samples

No field-collected animals were used in this study.

Ethics oversight

No ethical approval is required for Caenorhabditis elegans studies.

Note that full information on the approval of the study protocol must also be provided in the manuscript.

Plants

Seed stocks

No plants or seed stocks were used in this study.

Novel plant genotypes

No plants or novel plant genotypes were used in this study.

Authentication

No plants were used in this study.
