# Supplementary material for: CYLD-mediated lysine63 deubiquitination regulates synaptic transmission and autophagy to mitigate age-related sequelae
Source: Nat Commun. 2026 Jun 4;17:7164. doi: 10.1038/s41467-026-73966-5 (PMC13396389; doi:10.1038/s41467-026-73966-5)
Supplement: Supplementary file 5 — Transparent Peer Review file [file 41467_2026_73966_MOESM5_ESM.pdf]

nature portfolio

Peer Review File

CYLD-mediated lysine63 deubiquitination regulates synaptic transmission and autophagy to mitigate age-related sequelae

Corresponding Author: Professor Nektarios Tavernarakis

This file contains all reviewer reports in order by version, followed by all author rebuttals in order by version.

Version 0:

Reviewer comments:

Reviewer #1

(Remarks to the Author)
Summary
In this manuscript, Sotiriou and colleagues set out to evaluate the role of CYLD in neuronal aging in C. elegans. As there is only one CYLD homolog in C. elegans, namely CYLD-1, analyses of its function are not complicated by redundant paralogous genes. The authors conclude that DUB catalytic activity of CYLD-1 is required for learning, synaptic homeostasis and neurotransmission. The authors further conclude that CYLD-1 is necessary to preserve motor neuron homeostasis sustaining locomotory capacity and to support autophagy-lysosomal pathway in neurons and peripheral tissues. Overall, the topic is interesting and many of the data are rather intriguing. The authors have provided solid evidence on CYLD-1 preservation of synaptic function. However, several major conclusions lack sufficient characterization or mechanistic understanding. As examples, lack of characterization of the relationship of CYLD-1 to known longevity paradigms and failure to delineate the possibilities of intrinsic, cell autonomous functions of CYLD-1 from extrinsic, cell non-autonomous functions make this study overly preliminary in its current form.

Major issues

1. CYLD-1 promotes longevity (Fig 1)
The authors conclude that two alleles of cyld-1, namely cyld-1(tm3768) and cyld-1(ok3637), had similar reductions in lifespan (Line 108). However, the aging data show a large disparity between the 2 alleles with cyld-1(ok3637) showing minor impact on lifespan. In addition, because the authors observe that loss of cyld-1 compromises life extension of daf-2 mutants, they conclude cyld-1 is required to extend lifespan. Without testing either another paradigm of life extension or determine how or even if the insulin signaling pathway targets cyld-1, this standalone observation has many possible interpretations beyond the conclusion that cyld-1 is required to extend lifespan. Moreover, the conclusion that neuronal CYLD-1 activity is sufficient to promote animal longevity (Line 167) is preliminary and based on one RNAi experiment. The authors state that RNAi was able to show that neuronal CYLD-1 is sufficient for shortened lifespan (Line 167). However, RNAi (a depletion method) tests for necessity and not sufficiency. Understanding these differences are essential for making a conclusion on longevity rather than homeostasis, viability, or tissue integrity, among other possibilities. This point becomes even more critical when evaluating the autophagy / lysosome contributions (more comments below).

2. CYLD-1 promotes autophagic flux and modulates and lysosomal morphology (Figs 5-6)
Relationship of CYLD-1 to autophagy / lysosomal pathways in support of nerve function is unclear with the authors' current findings. As an example, the ced-1p::nuc-1::mCherry reporter itself only marks epidermal lysosomes but does not mark lysosomes of any other tissues. However, the authors conclude that lysosome function is impacted by CYLD-1 and therefore important to nerve phenotypes. While the impact of epidermal lysosome homeostasis to nerve function is intriguing, the authors have not determined the connection of epidermal lysosomes to nerves and whether in fact the regulation is cell autonomous (intrinsic regulation) or cell non-autonomous (extrinsic). Based on their data, the authors can only conclude that cyld-1 impacts nerve function and epidermal lysosomes but it is unclear how or whether these observations are connected.

3. CYLD-1 functions as a K63-specific DUB in the nervous system (Fig 2)
Inadequate characterization of CYLD-1 expression. With a single animal shown, the authors have no way to delineate if the mosaic expression they observe for cycl-1::mCherry from pharynx to various neurons is reflective of the innate biology of cyld-1 or the transgene itself. Additionally, the authors also suggest that CYLD-1 is expressed in the intestine but the signal

looks more consistent with autofluorescence from lysosome related organelles and other vesicles. The autoflourescence phenomenon is well known and requires a basic “no transgene control”. Given approximately 300 neurons in C. elegans and no large-scale mosaic analysis performed by the authors, including proper controls, nothing conclusive about expression patterns can be made at this point.

4. CYLD-1 confers neuroprotection during ageing (Fig 3)
Inadequate characterization of commissural defects. The authors only analyzed the cyld-1(tm3768) allele for defects and should at a minimum analyze this phenotype with the other cyld-1(ok3637) allele which showed considerable differences in aging.

Reviewer #2

(Remarks to the Author)
This study investigates roles of CYLD-1, a lysine63 specific deubiquitinase, in C. elegans neuronal cells during aging. The authors show CYLD-1 and its deubiquitinase activity are critical for the maintenance of normal lifespan. In addition, they demonstrate CYLD-1 deubiquitinase activity is essential for the preservation of neuronal physiology including locomotive and cognitive function. They further show CYLD-1 is required for autophagic activity and maintenance of lysosomal homeostasis. The study documents interesting facts regarding the function of CYLD-1, but the mechanistic link among K63 deubiquitination, the synaptic transmission and autophagy-lysosome system is unclear. Whether these are all connected or not needs to be experimentally addressed. The followings are specific comments for the authors.

Major
In line 94, the authors stated “Mechanistically, CYLD-1 exerts its effects on neuronal function and lifespan by promoting autophagic recycling and lysosomal homeostasis in the nervous system and other tissues.”, but this is not supported by the experiment. Is autophagic defect by CYLD-1 mutation a main cause for the shortevity and neuronal defect? If so, the authors need to show the underlying mechanism and their connection. Knockdown of genes involved in autophagy (bec-1, unc-51 etc.) recapitulates the defect observed (lifespan, neurotransmission and cognitive functions etc.) in CYLD-1 deletion and AS mutants? Cyld-1 AS mutants also show autophagic defect, abnormal autolysosomal and lysosomal morophology? The author needs to show if there are specific target protein of K63 deubiquitination by CYLD-1 regulating both neuronal functions and autophagy.

The authors showed that neuronal RNAi of cyld-1 is sufficient to reduce lifespan. However, the role of neuronal cyld-1 in aging is still unclear. Is neuronal WT cyld-1 but not AS mutant able to restore the lifespan of cyld-1 deletion mutants?

In Fig.1, Fig. 4C-E, it is hard to distinguish the lines between WT and cyld-1 mutants/ RNAi. One of them should be changed to color lines or dashed lines.

How does cyld-1 regulate autophagy in the hypodermis? Cell autonomously or non-cell autonomously via neurotransmitters or via neuropeptides? The authors showed neuronal roles of cyld-1, but did not demonstrate the mechanism of neuron-peripheral interaction in regulation of autophagy in hypodermis (Fig. 6G).

Minor
In Figure 1A, the survival curve of WT looks at 25C, but not at 20C. The author should confirm the experimental temperature of this assay. If the authors performed the survival assay at 25C, the exact experimental temperatures should be mentioned in the methods section or in the figure legend.

Reviewer #3

(Remarks to the Author)
The manuscript by Sotiriou et al. investigates the role of the deubiquitinase CYLD-1 in regulating lifespan and aging-related physiology. The authors report that aging related physiology such as synapse function and learning and memory are compromised in the cyld-1 mutants. Besides, autophagy function and lysosome morphology are altered in cyld-1 mutants and propose that CYLD-1 regulates aging through autophagy. However, the data provided do not sufficiently support these conclusions. The most critical issue is the lack of mechanistic insight—specifically, the failure to identify a direct K63-linked deubiquitination target of CYLD-1 that connects its function to aging. Instead, the study primarily describes phenotypic observations without establishing a cause-and-effect relationship. It is also unclear whether the reported synaptic and lysosomal defects are primary effects of CYLD-1 loss or merely secondary consequences of accelerated aging in the mutants.
1. Identification of CYLD-1’s K63-deubiquitination target is missing. The study does not establish which specific substrate(s) CYLD-1 acts on to regulate synaptic function, autophagy, or lysosomal morphology. Without this, it is difficult to determine how CYLD-1 mechanistically contributes to aging.
2. The evidence supporting CYLD-1 as a K63 deubiquitinase is weak. The polyubiquitin blot (Figure S1B) shows only a subtle increase in ubiquitination, and the loading control (a-tubulin) is inconsistent across lanes, making it difficult to interpret the data conclusively.

3. The genetic interaction analysis with daf-2 is problematic. The data in Figure 1C-D indicate that cyld-1 and daf-2 act in parallel pathways rather than in a single genetic pathway. The observed effects could simply be additive rather than mechanistically linked.
4. The explanation of paralysis phenotypes (Figure 4C) is unclear. The authors conclude that paralysis is due to increased cholinergic transmission, but the alternative possibility of decreased GABAergic transmission is not addressed, even though compromised GABAergic synapse formation was observed in the mutants.
5. The colocalization data in Figure 4A are not convincing. The quantification lacks clarity, and it is unclear how colocalization was measured and analyzed.
6. The criteria for defining neurodegeneration (Figure 3) are not well explained. There is no clear description of how neurodegeneration was quantified, and no supporting references are provided to justify the criteria used.
7. Allelic differences between cyld-1(tm3769) and cyld-1(ok3637) are not addressed. The reduced lifespan is more pronounced in the tm3769 allele, yet the differences between the two alleles are not explained. Additional data on the ok3637 allele should be included in Figure S1 for clarity.

Version 1:

Reviewer comments:

Reviewer #1

(Remarks to the Author)
In this revised manuscript, Sotiriou and colleagues have now convincingly established a role for CYLD-1 catalytic activity in aging. The authors have made concerted efforts to bolster their conclusions with substantial and well-controlled experiments. As examples, they have clarified the necessity rather than sufficiency of cyld-1 in aging. They have provided two additional paradigms of aging analyses. The authors have further clarified their findings in both autophagic flux and neuronal expression of CYLD-1. Finally, the authors have supported with outcrossed alleles the neuroprotective role of CYLD-1 in aging. I congratulate the authors on a clear study. They have addressed all of my critiques and I have no further objection.

Reviewer #2

(Remarks to the Author)
The authors responded to all of my concerns I raised for the original manuscript in a satisfactory manner and the revised manuscript is acceptable for publication.

Reviewer #3

(Remarks to the Author)
The manuscript by Sotiriou et al. has been substantially improved in the revised version, particularly with respect to clarifying the genetic pathways involving cyld-1 and strengthening the analyses related to autolysosomal/lysosomal function. However, several concerns still need to be addressed before the manuscript can be accepted.

1.Have the authors tested whether systemic or neuron-specific overexpression of cyld-1 is sufficient to extend lifespan and/or preserve learning and memory during aging? Such gain-of-function experiments would help establish whether cyld-1 is not only necessary but also sufficient for promoting longevity and cognitive resilience.

2.Figure 1E–G: Revise the interpretation of these data. The results indicate that cyld-1 is not required for the lifespan extension conferred by daf-2, eat-2, or glp-1, and conversely that these longevity interventions do not substantially modify the shortened lifespan phenotype of cyld-1 loss-of-function animals. This apparent lack of epistasis is more consistent with cyld-1 acting in a parallel or largely independent pathway from IIS, dietary restriction, and germline signaling.

In addition, for Figure 1G, it is unclear whether there is a statistically significant difference between cyld-1(RNAi) and glp-1; cyld-1(RNAi). If there is no significant difference, this would instead support the interpretation that cyld-1 functions in the same pathway as glp-1, with glp-1 being epistatic to cyld-1. Please provide the relevant statistical comparisons (including exact p values) and clarify the conclusion accordingly.

3.Figure 4F–H: The interpretation of these data needs to be revised. While the unc-47 promoter–driven SNB-1 puncta density and fluorescence intensity appear decreased in cyld-1 mutants, the authors conclude that “these observations suggest that cyld-1–deficient animals preserve intact GABAergic neurotransmission despite their phenotypic alterations in GABAergic synapses (Fig. 4F–H).” This statement seems overstated given the observed reductions. Please clarify whether these changes are statistically significant, and if so, discuss the possibility that GABAergic presynaptic function may be partially impaired, rather than fully intact. If the differences are not significant, the authors should explicitly state this and temper the conclusion accordingly.

4.Figure 5I: The reported statistics appear inconsistent with the plotted data. The confidence intervals for lgg-2 and cyld-1; lgg-2 appear non-overlapping, yet the authors indicate that the comparison is not significant. Please clarify the statistical test used (including exact p values and any multiple-comparison correction, if applicable), and ensure that the confidence intervals and significance annotations are presented consistently.

Version 2:

Reviewer comments:

Reviewer #3

(Remarks to the Author)
The authors have addressed all of my concerns.

Open Access This Peer Review File is licensed under a Creative Commons Attribution 4.0 International License, which permits use, sharing, adaptation, distribution and reproduction in any medium or format, as long as you give appropriate credit to the original author(s) and the source, provide a link to the Creative Commons license, and indicate if changes were made.
In cases where reviewers are anonymous, credit should be given to 'Anonymous Referee' and the source.
The images or other third party material in this Peer Review File are included in the article's Creative Commons license, unless indicated otherwise in a credit line to the material. If material is not included in the article's Creative Commons license and your intended use is not permitted by statutory regulation or exceeds the permitted use, you will need to obtain permission directly from the copyright holder.
To view a copy of this license, visit https://creativecommons.org/licenses/by/4.0/

December 27, 2025

Ref: NCOMMS-25-03716-T

Dear Reviewers,

We would like to thank you for your time and effort in reviewing our manuscript. We have carefully considered each of the points raised and made every possible effort to address them experimentally. In doing so, we strived to include additional information and data within the text and Figures as well as in the Supplementary Figures. All changes made in the revised manuscript are highlighted.

We believe that, with your encouraging and constructive input, we have been able to resubmit a much stronger and more comprehensive report. A point-by-point response to each comment follows below (shown in **blue**).

We will be standing by for any further clarifications or information, pertinent to our final version that may be required. Thank you very much again, for your consideration of our manuscript.

Sincerely,

Nektarios Tavernarakis,

for the authors

Reviewer #1:

In this manuscript, Sotiriou and colleagues set out to evaluate the role of CYLD in neuronal aging in C. elegans. As there is only one CYLD homolog in C. elegans, namely CYLD-1, analyses of its function are not complicated by redundant paralogous genes. The authors conclude that DUB catalytic activity of CYLD-1 is required for learning, synaptic homeostasis and neurotransmission. The authors further conclude that CYLD-1 is necessary to preserve motor neuron homeostasis sustaining locomotory capacity and to support autophagy-lysosomal pathway in neurons and peripheral tissues. Overall, the topic is interesting and many of the data are rather intriguing. The authors have provided solid evidence on CYLD-1 preservation of synaptic function.

R1_0.1

We thank the Reviewer for the positive summary and appreciation of our work.

However, several major conclusions lack sufficient characterization or mechanistic understanding. As examples, lack of characterization of the relationship of CYLD-1 to known longevity paradigms and failure to delineate the possibilities of intrinsic, cell autonomous functions of CYLD-1 from extrinsic, cell non-autonomous functions make this study overly preliminary in its current form.

R1_0.2

We thank the Reviewer for raising these two points. We have experimentally deciphered both topics in our revised manuscript. Briefly, we demonstrate a generalized role of CYLD-1 in distinct longevity

paradigms beyond perturbed insulin signalling, i.e., calorie restriction and elimination of the germline stem cells. We further provide evidence that neuronal CYLD-1 regulates autophagic-lysosome homeostasis in neurons without affecting hypodermal autophagic-lysosome physiology in a cell non-autonomous manner.

1. CYLD-1 promotes longevity (Fig 1)

The authors conclude that two alleles of cyld-1, namely cyld-1(tm3768) and cyld-1(ok3637), had similar reductions in lifespan (Line 108). However, the aging data show a large disparity between the 2 alleles with cyld-1(ok3637) showing minor impact on lifespan.

R1_1.1

We thank the Reviewer for pointing out this issue. We have obtained the RB2610 strain with the *ok3637* variation from the *Caenorhabditis* Genetics Center (CGC). First, this variation was not placed on the physical map and its exact coordinates were unknown. CGC mentions that it comprises a deletion of approximately 900bp included in the area of chrIII:9911553-9912787. We have mapped the exact type and site of mutation, and now report a deletion of 858bp, spanning the area chrIII:9911587-9912444 (new Fig. 1A). This deletion removes the USP domain and specifically a part of exon 14 that includes the catalytic cysteine 774, the whole exon 15 and a part of exon 16. At the protein level, the above results in the deletion of the residues 715-960 and shift in the reading frame and a stop codon after 13 residues (-VKHAQKSTARRVS*). Second, since the exact type of mutation was unknown, the strain RB2610 was not outcrossed. We have outcrossed the RB2610 strain twice with laboratory wild type N2 animals resulting in the strain IR3371. We have utilized the IR3371 strain and repeated the lifespan assays, as well as neurodegeneration assays (see also comment R1_4 for results regarding neurodegeneration) and mTOR activity monitoring (Fig. 5K, L). The outcrossed strain shows identical lifespan reduction to the one with the *cyld-1(tm3768)* variation (new Fig. 1B). It worth to mention that the previous results from the two lines of the CRISPR-Cas9-generated catalytic dead *cyld-1* mutant strains demonstrated similar lifespan reduction highlighting the role of CYLD-1’s DUB activity in lifespan regulation (initial Fig. 1G, incorporated in new Fig. 1B). In addition, *cyld-1(RNAi)* shortens lifespan in wild type animals (initial Fig. 1C, new Fig. 1D, F, G). Finally, we also note here that since the *tm3768* variation of *cyld-1* (strain TM3768) was known, the strain TM3768 was already outcrossed three times (strain IR2847) as mentioned in the Table S1.

In addition, because the authors observe that loss of cyld-1 compromises life extension of daf-2 mutants, they conclude cyld-1 is required to extend lifespan. Without testing either another paradigm of life extension or determine how or even if the insulin signaling pathway targets cyld-1, this standalone observation has many possible interpretations beyond the conclusion that cyld-1 is required to extend lifespan.

R1_1.2

We thank the Reviewer for this suggestion and we agree. We have now included two additional paradigms of lifespan extension, these of *eat-2(ad465)* and *glp-1(e2141)*, representing calorie restriction and reproductive curtailment respectively. In both paradigms, depletion of *cyld-1* shortens lifespan (new Fig. 1F, G).

Moreover, the conclusion that neuronal CYLD-1 activity is sufficient to promote animal longevity (Line 167) is preliminary and based on one RNAi experiment. The authors state that RNAi was able to show that neuronal CYLD-1 is sufficient for shortened lifespan (Line 167). However, RNAi (a depletion method) tests for necessity and not sufficiency. Understanding these differences are essential for making a conclusion on longevity rather than homeostasis, viability, or tissue integrity, among other possibilities. This point becomes even more critical when evaluating the autophagy / lysosome contributions (more comments below).

R1_1.3

We thank the Reviewer for improving the clarity of our work with these suggestions. Our conclusion that neuronal CYLD-1 activity is necessary (now corrected) to promote animal longevity is based on multiple experimental repeats, performed with two independent neuronal RNAi-competent strains, MAH677 and TU3401. The first rescues *sid-1* expression utilizing the neuronal specific *rgef-1* promoter in *sid-1(qt9)* loss of function mutant animals while the second rescues *sid-1* expression utilizing the neuronal specific *unc-119* promoter in *sid-1(pk3321)* (corresponding to a residue substitution) mutant animals. We attempted to restore the *cyld-1* expression specifically in neurons in the *cyld-1* mutant animals to examine whether animal longevity is rescued (similar to global rescue under the *cyld-1* promoter presented in new Fig. 1C). However, expression of *cyld-1* under the neuronal-specific promoter *rab-3* caused adverse effects such as developmental delay, internal hatching and compromised locomotion. We speculate that the developmental pattern of expression, the strength of the promoter (Fig. R1_1.3, please see the aggregation pattern at the lower panel) or the ectopic expression in additional neurons elicited these deficits. Finally, we have adapted our conclusions here and throughout the manuscript to reflect the necessity tested by the depletion approaches.

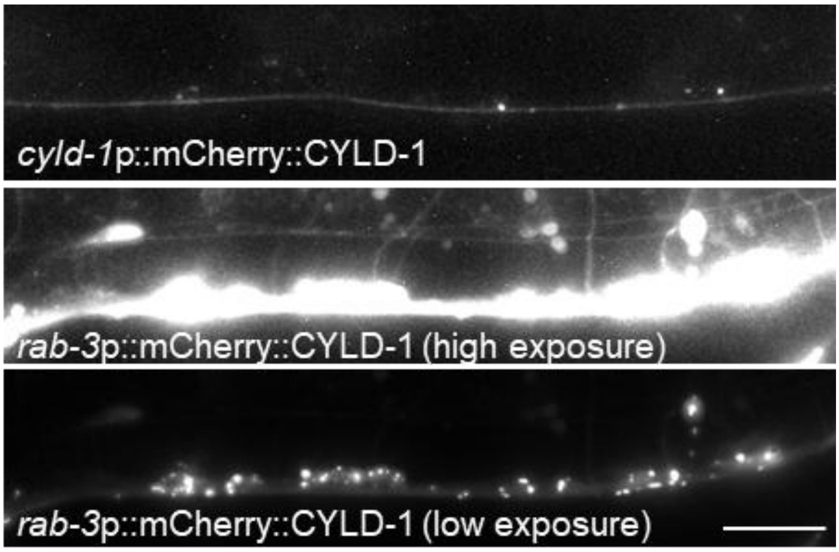

Figure R1_1.3: Transgenic animals expressing mCherry::CYLD-1 under the control of the endogenous (upper panel) or the pan-neuronal promoter rab-3 (middle panel at high exposure and lower panel at low exposure). Images in the upper and middle panel were obtained using the same exposure settings, illustrating a stronger expression or retention of mCherry(::CYLD-1) under the control of the rab-3 promoter. Scale bar, 20μm.

2. CYLD-1 promotes autophagic flux and modulates and lysosomal morphology (Figs 5-6)

Relationship of CYLD-1 to autophagy / lysosomal pathways in support of nerve function is unclear with the authors' current findings. As an example, the ced-1p::nuc-1::mCherry reporter itself only marks epidermal lysosomes but does not mark lysosomes of any other tissues. However, the authors conclude that lysosome function is impacted by CYLD-1 and therefore important to nerve phenotypes. While the impact of epidermal lysosome homeostasis to nerve function is intriguing, the authors have not determined the connection of epidermal lysosomes to nerves and whether in fact the regulation is cell autonomous (intrinsic regulation) or cell non-autonomous (extrinsic). Based on their data, the authors can only conclude that cyld-1 impacts nerve function and epidermal lysosomes but it is unclear how or whether these observations are connected.

R1_2

We thank the Reviewer for this comment and for the chance to provide independent experimental evidence to elucidate the possible tissue-specific roles of CYLD-1. To elucidate the role of CYLD-1 in the autophagic-lysosome pathway, we have investigated autophagosome and autolysosome number and morphology in neurons of the nerve ring in *cyld-1* mutant animals (Fig. 5A, B and 6D-F). *cyld-1* mutant

animals exhibit an increase in the number of autophagosomes, as well as an increase in both the number and the length of autolysosomal tubules in neurons. To extend our study in a relevant neuronal subpopulation, we monitored the lysosomal morphology in animals expressing the lysosomal membrane marker CTNS-1::mCherry in cholinergic neurons of the ventral nerve cord (strain KG2430). CYLD-1 deficiency causes increased lysosomal length in cholinergic neurons (new Fig. 7H, I), consistent with our previous results concerning the autophagosomal and autolysosomal phenotype upon *cyld-1* deficiency in neurons of the nerve ring (Fig. 5A, B and 6D-F). We have also monitored the lysosomal morphology in *cyld-1* deficient animals expressing the same lysosomal membrane marker CTNS-1, endogenously tagged with wrmScarlet (strain PHX5270). CYLD-1 deficiency in these animals induces the formation of lysosomal network in hypodermis (new Fig. 7C-E). Since CYLD-1 expression is not observed, at least in fluorescent observable levels, in the hypodermal tissue, there may be indeed a cell non-autonomous function of neuronal CYLD-1 in hypodermal lysosomes that we had not investigated in our initial study.

While we have not connected the function of CYLD-1 in hypodermal lysosome homeostasis to nerve function in a cell non-autonomous manner, the Reviewer gave us the opportunity to examine whether neuronal CYLD-1 affects cell non-autonomously the physiology of hypodermal lysosomes. We compared the impact of systemic to neuronal-specific RNAi-mediated depletion of CYLD-1 in autolysosomal morphology of hypodermis. Systemic depletion of CYLD-1 results in increased autolysosomal (and lysosomal) network formation in hypodermis as evidenced previously (initial Fig. 6G-L and S3C-I or new Fig. 6G-J; 7A-E, J; S3C-I), while neuronal-specific depletion of CYLD-1 does not affect the morphology of hypodermal autolysosomes (new Fig. 7J). These results indicate that neuronal CYLD-1 regulates neuronal but not hypodermal autolysosome physiology in a cell non-autonomous manner.

3. CYLD-1 functions as a K63-specific DUB in the nervous system (Fig 2)

Inadequate characterization of CYLD-1 expression. With a single animal shown, the authors have no way to delineate if the mosaic expression they observe for cycl-1::mCherry from pharynx to various neurons is reflective of the innate biology of cyld-1 or the transgene itself. Additionally, the authors also suggest that CYLD-1 is expressed in the intestine but the signal looks more consistent with autofluorescence from lysosome related organelles and other vesicles. The autofluorescence phenomenon is well known and requires a basic “no transgene control”. Given approximately 300 neurons in C. elegans and no large-scale mosaic analysis performed by the authors, including proper controls, nothing conclusive about expression patterns can be made at this point.

R1_3

We thank the Reviewer for raising these points and we agree. In our initial submission, we have not presented whole worm imaging data or imaging data from different lines. Characterization of CYLD-1 expression was based on both *cyld-1p::mcherry* (initial Fig. 2A) and *cyld-1p::mcherry::cyld-1* (initial Fig. S2A) animals. We now include whole worm imaging of one *cyld-1p::mcherry* line and four *cyld-1p::mcherry::cyld-1* lines (new Fig. S1A, B). Several transgenic worms are compared with wild type (N2) animals as no transgene control, in two different exposures. Both transgenic strains and all lines display identical expression pattern in pharynx, neurons (nerve ring and ventral nerve cord) and intestine, albeit expression level differences exist among independent lines. Please compare the respective high exposure images to discriminate the punctate intestinal autofluorescence pattern of wild type control animals with the diffused intestinal pattern upon *cyld-1* promoter-induced expression in the transgenic worms. We have further demonstrated that *cyld-1* is expressed in cholinergic and GABAergic neurons of the ventral nerve cord (Fig. 2B and 4B).

4. CYLD-1 confers neuroprotection during ageing (Fig 3)

Inadequate characterization of commissural defects. The authors only analyzed the cyld-1(tm3768) allele for defects and should at a minimum analyze this phenotype with the other cyld-1(ok3637) allele which showed considerable differences in aging.

R1_4

We thank the Reviewer for this comment. We have now analyzed the neuronal defects during ageing using the outcrossed *cyld-1(ok3637)* strain as well. The results show a similar degree of neurodegeneration upon ageing compared to *cyld-1(tm3768)* animals (new Fig. 3A-D). We further included the *cyld-1(ok3637)* mutants to monitor motor neuron loss upon ageing. Again, both *cyld-1* variants exhibit similar loss of cholinergic and GABAergic neurons in old animals (new Fig. 3E, F).

Reviewer #2:

This study investigates roles of CYLD-1, a lysine63 specific deubiquitinase, in C. elegans neuronal cells during aging. The authors show CYLD-1 and its deubiquitinase activity are critical for the maintenance of normal lifespan. In addition, they demonstrate CYLD-1 deubiquitinase activity is essential for the preservation of neuronal physiology including locomotive and cognitive function. They further show CYLD-1 is required for autophagic activity and maintenance of lysosomal homeostasis. The study documents interesting facts regarding the function of CYLD-1, but the mechanistic link among K63 deubiquitination, the synaptic transmission and autophagy-lysosome system is unclear. Whether these are all connected or not needs to be experimentally addressed. The followings are specific comments for the authors.

R2_0

We thank the Reviewer for concluding that our study presents interesting findings. We highly appreciate the suggestions to improve our work which have increased the overall coherence of our study.

In line 94, the authors stated “Mechanistically, CYLD-1 exerts its effects on neuronal function and lifespan by promoting autophagic recycling and lysosomal homeostasis in the nervous system and other tissues.”, but this is not supported by the experiment. Is autophagic defect by CYLD-1 mutation a main cause for the shortevity and neuronal defect? If so, the authors need to show the underlying mechanism and their connection. Knockdown of genes involved in autophagy (bec-1, unc-51 etc.) recapitulates the defect observed (lifespan, neurotransmission and cognitive functions etc.) in CYLD-1 deletion and AS mutants? Cyld-1 AS mutants also show autophagic defect, abnormal autolysosomal and lysosomal morophology? The author needs to show if there are specific target protein of K63 deubiquitination by CYLD-1 regulating both neuronal functions and autophagy.

R2_1

We thank the Reviewer for urging us to further dissect the interplay between CYLD-1 and autophagy. We expanded our analysis of *cyld-1* deficiency as suggested to evaluate the role of autophagy beyond lifespan i.e. in neurotransmission as well as in cognitive functions. In Fig. 5G, H we show that knockdown of the autophagy gene *unc-51* or mutant animals for the autophagy gene *lgg-2* display shortened lifespan compared to wild type animals and furthermore they do not exacerbate the lifespan shortening of the *cyld-1* mutant animals. We further investigated the role of autophagy in neurotransmission (utilizing WMicrotracker ONE, an automated system that provides large-scale quantitative worm activity measurements) and animal cognition (short-term associative memory) upon *cyld-1* deficiency. We demonstrate that inhibition of autophagy through depletion of the core autophagic gene *lgg-2*, recapitulates the defects observed upon *cyld-1* deficiency (new Fig. 5I, J). Moreover, combined depletion of *cyld-1* and *lgg-2* neither exacerbates aldicarb-mediated paralysis nor aggravates compromised short-term associative memory in chemotaxis assay towards isoamyl alcohol of *cyld-1* deficient worms (new Fig. 5I, J). These results indicate that autophagy and CYLD-1 likely function in the same pathway to promote longevity, as well as support neurotransmission and short-term associative memory.

Both *cyld-1* deletion (*tm3768*) and catalytic dead (*as1*) mutant animals demonstrated impaired acidification of intestinal lysosomes, underscoring the critical role of CYLD-1 catalytic function in maintaining lysosomal homeostasis in these cells (initial Fig. S3J, K or new Fig. 7F, G). We now show that

similar to *cyld-1* deletion (*tm3768*) mutants, the catalytically inactive *cyld-1*(*as1*) animals demonstrate increased autolysosomal network formation in hypodermis compared to wild type worms, underscoring the critical role of CYLD-1 catalytic function in maintaining a functional autophagic-lysosome pathway in this tissue as well (new Fig. 6G-J and new Fig. S3D-F). Additionally, we examined whether the activity of the upstream master regulator of cellular metabolism and inhibitor of autophagy, LET-363/mTOR, is affected upon *cyld-1* deficiency. *cyld-1* deficiency leads to LET-363/mTOR signalling activation, judging from the increased phosphorylation levels of RSKS-1, the *C. elegans* ortholog of p70-S6 kinase, a substrate of mTOR (new Fig. 5K, L). Please refer to response R3_1 as well. Finally, while beyond the scope of this manuscript, we agree that identification of CYLD-1 substrate(s) would indeed shed light in the CYLD-1 mode of action in autophagy and its impact on neuronal functions. Nevertheless, this is a complete separate study, in itself, which we plan to pursue in the future.

The authors showed that neuronal RNAi of cyld-1 is sufficient to reduce lifespan. However, the role of neuronal cyld-1 in aging is still unclear. Is neuronal WT cyld-1 but not AS mutant able to restore the lifespan of cyld-1 deletion mutants?

R2_2

We thank the Reviewer for proposing this experiment. As described in the comment R1_1.3, we attempted to restore the *cyld-1* expression specifically in neurons in the *cyld-1* deletion mutants to examine whether animal longevity is rescued. Unfortunately, expression of *cyld-1* under the neuronal-specific promoter *rab-3* caused adverse effects such as developmental delay, internal hatching and compromised locomotion. We speculate that the developmental pattern of expression, the strength of the promoter (Fig. R1_1.3, please see the aggregation pattern at the lower panel) or the ectopic expression in additional neurons elicited these deficits. Nevertheless, our results illustrate that neuronal depletion of *cyld-1* causes reduced lifespan (Fig. 2E and Fig. S1E). Furthermore, we now demonstrate with additional *cyld-1* deletion mutant animals that CYLD-1 preserves motor neuron integrity and maintains locomotory capacity upon ageing (new Fig. 3).

In Fig.1, Fig. 4C-E, it is hard to distinguish the lines between WT and cyld-1 mutants/ RNAi. One of them should be changed to color lines or dashed lines.

R2_3

We thank the Reviewer for improving the presentation of our results. We have now used a higher contrast gray line for *cyld-1* mutants/*RNAi* in all figure panels.

How does cyld-1 regulate autophagy in the hypodermis? Cell autonomously or non-cell autonomously via neurotransmitters or via neuropeptides? The authors showed neuronal roles of cyld-1, but did not demonstrate the mechanism of neuron-peripheral interaction in regulation of autophagy in hypodermis (Fig. 6G).

R2_4

We thank the Reviewer for identifying and mentioning this flaw as Reviewer 1 did (R1_2 second part). Our previous and current observations do not support a CYLD-1-mediated neuron-hypodermis interaction. Indeed, since CYLD-1 expression is not observed, at least in fluorescent observable levels, in the hypodermal tissue, there may be a cell non-autonomous function of CYLD-1 in hypodermal lysosomes. To test whether such regulation stems from neuronal CYLD-1, we investigated whether neuronal-specific depletion of CYLD-1 affects the morphology of hypodermal autolysosomes (new Fig. 7J). In contrast to systemic *cyld-1(RNAi)*, the architecture of the hypodermal autolysosome network remains unaffected upon depletion of neuronal CYLD-1, suggesting that neuronal CYLD-1 does not affect hypodermal autolysosome physiology in a cell non-autonomous manner. We have revised our manuscript accordingly to avoid such an assumption.

In Figure 1A, the survival curve of WT looks at 25C, but not at 20C. The author should confirm the experimental temperature of this assay. If the authors performed the survival assay at 25C, the exact experimental temperatures should be mentioned in the methods section or in the figure legend.

R2_5

We thank the Reviewer for this observation. All lifespan assays were performed in 20°C. We attribute this curve discrepancy in intra-lab experimental variations, accounting possibly for subtle alterations in OP50 concentration, NGM consistency/humidity or ambient temperature. We have replaced this experiment with another replication that demonstrates identical observations and statistical significance to the initial one.

Reviewer #3:

The manuscript by Sotiriou et al. investigates the role of the deubiquitinase CYLD-1 in regulating lifespan and aging-related physiology. The authors report that aging related physiology such as synapse function and learning and memory are compromised in the cyld-1 mutants. Besides, autophagy function and lysosome morphology are altered in cyld-1 mutants and propose that CYLD-1 regulates aging through autophagy. However, the data provided do not sufficiently support these conclusions. The most critical issue is the lack of mechanistic insight—specifically, the failure to identify a direct K63-linked deubiquitination target of CYLD-1 that connects its function to aging. Instead, the study primarily describes phenotypic observations without establishing a cause-and-effect relationship.

R3_0.1

We thank the Reviewer for the constructive criticism that strengthened our study. We have extended our analysis of the role of *cyld-1* in *C. elegans* longevity to two additional paradigms of lifespan extension, these of *eat-2(ad465)* and *glp-1(e2141)*, representing calorie restriction and reproductive curtailment respectively. In both paradigms, depletion of *cyld-1* shortens lifespan (new Fig. 1F, G). Knockdown of the autophagy gene *unc-51* or mutant animals for the autophagy gene *lgg-2* display shorter lifespan than wild type animals and furthermore they do not exacerbate the lifespan shortening of the *cyld-1* mutant animals (Fig. 5G, H). In addition, we expanded our analysis of *cyld-1* deficiency to evaluate the role of autophagy in the observed deficits in neurotransmission and cognitive functions (short-term associative memory). We demonstrate that inhibition of autophagy through deletion of the core autophagic gene *lgg-2*, recapitulates the defects observed upon *cyld-1* deficiency (new Fig. 5I, J). Moreover, combined deletion of *cyld-1* and *lgg-2* neither exacerbates aldicarb-mediated paralysis nor aggravates compromised short-term associative memory in chemotaxis assay towards isoamyl alcohol of *cyld-1* deficient worms (new Fig. 5I, J). These results indicate that autophagy and CYLD-1 likely function in the same pathway to promote longevity, as well as support neurotransmission and short-term associative memory. As mentioned above, we respectfully note that identification of a direct K63-linked deubiquitination target(s) of CYLD-1 is beyond the scope of the current manuscript. Nevertheless, this is a complete separate study, in itself, which we plan to pursue in the future.

It is also unclear whether the reported synaptic and lysosomal defects are primary effects of CYLD-1 loss or merely secondary consequences of accelerated aging in the mutants.

R3_0.2

We thank the Reviewer for raising this possibility. The synaptic (Fig. 4A-H and new S2B-E) and lysosomal (new Fig. 6 and S3) defects that we observed, take place in day one adult animals. This time point is commonly considered as a breakpoint between the larval development stages and ageing or as the onset of adulthood. We hypothesize that since our observations take place early enough in *C. elegans* lifecycle, an accelerated ageing-related effect would be extremely constricted temporally. However, we tested day one

animals for traits that manifest during physiological ageing or resemble premature ageing conditions. We compared autofluorescence derived from age pigment of gut granules (10.1016/0047-6374(77)90043-4, 10.1016/j.xpro.2023.102285) as well as mitochondrial network physiology (10.1016/j.mad.2006.07.002, 10.1016/j.cell.2022.11.001) between wild type and *cyld-1* deficient animals. Both wild type and *cyld-1* deficient animals show age-matched intestinal autofluorescence and intact mitochondrial physiology (Fig. R3_0.2). In addition, assessment of locomotory ability through body bend rate (10.1016/j.xpro.2023.102285) which is strongly associated with mitochondrial structure in *C. elegans* (10.18632/aging.101654), further illustrates the physiological healthspan under *cyld-1* deficiency in day one animals (Fig. 3G, D1 comparison).

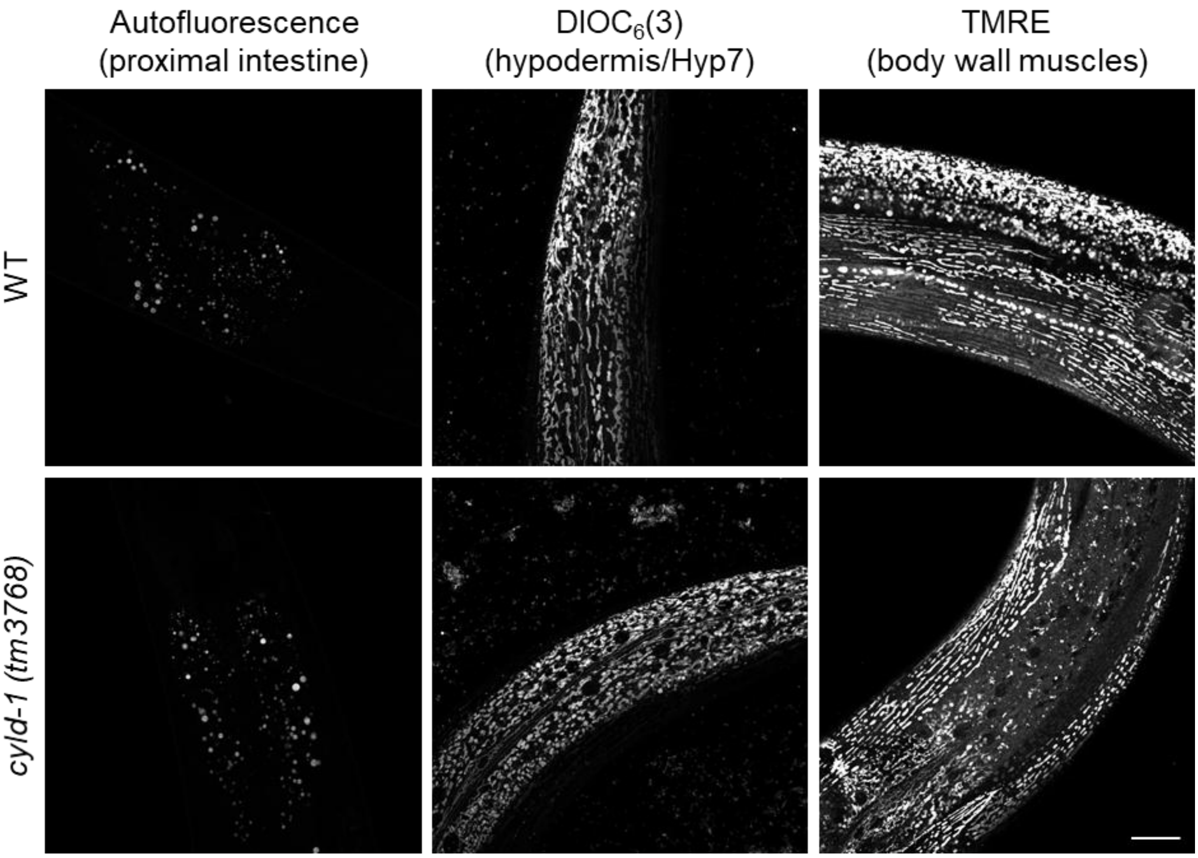

Fig. R3_0.2: Confocal microscopy imaging of wild type and cyld-1(tm3768) animals (autofluorescence and DIOC$_6$(3) at 488nm; TMRE at 561nm) at day one of adulthood. Staining with 100nM DIOC$_6$(3) and 200nM TMRE was performed 16h before imaging. Scale bar, 20μm.

1. Identification of CYLD-1's K63-deubiquitination target is missing. The study does not establish which specific substrate(s) CYLD-1 acts on to regulate synaptic function, autophagy, or lysosomal morphology. Without this, it is difficult to determine how CYLD-1 mechanistically contributes to aging.

R3_1

While identification of direct target(s) of CYLD-1 is beyond the scope of the current study, we examined whether the activity of the upstream master regulator of cellular metabolism and inhibitor of autophagy, LET-363/mTOR, is affected upon *cyld-1* deficiency. We monitored the S386 phosphorylation of the LET-363 substrate RSKS-1, corresponding to the conserved S371 phosphorylation of the mammalian p70-S6 kinase (10.7554/eLife.31268, 10.17912/micropub.biology.001520), an indicator of TOR activity (10.1038/s41556-018-0205-1, 10.1038/s41580-019-0199-y, 10.1038/s41392-023-01608-z). *cyld-1* mutant animals including the endogenous catalytically inactive ones exhibited increased phospho-RSKS-1 levels compared to wild type worms, suggesting that *cyld-1* deficiency leads to LET-363/mTOR signaling activation (new Fig. 5K, L). We note here that normalization of phospho-RSKS-1 levels was made using α-tubulin, due to the lack of a respective RSKS-1/p70-S6K antibody recognizing total RSKS-1 in *C. elegans*

(10.7554/eLife.31268, 10.17912/micropub.biology.001520). Such an analysis remains the most comparable readout to the respective mammalian TOR activity studies currently available. *rsks-1(ok1255)* mutants carrying a deletion that includes S386 were used as a negative control for the antibody specificity. Overall, the above and previous observations highlight the implication of CYLD-1 activity in regulating metabolic and organellar responses, upstream (increased LET-363/mTOR activity) and downstream (autolysosome/lysosome physiology) of autophagy.

2. The evidence supporting CYLD-1 as a K63 deubiquitinase is weak. The polyubiquitin blot (Figure S1B) shows only a subtle increase in ubiquitination, and the loading control (a-tubulin) is inconsistent across lanes, making it difficult to interpret the data conclusively.

R3_2

The Reviewer may have overlooked the wild type lane where the signal from the K63-linked poly-ubiquitin antibody is substantially lower than the three lanes corresponding to cyld-1 mutant animals. We have removed the redundant Fig. 1E and included the normalized to α-tubulin densitometry of the indicative tm3768 and as1 mutants across four repeats (new Fig. 1H, I). To preserve the control variance in the western blot densitometry graphs, the mean of independent normalized control values was set to one (as described in DOI: 10.1038/s41598-022-22294-x).

3. The genetic interaction analysis with daf-2 is problematic. The data in Figure 1C-D indicate that cyld-1 and daf-2 act in parallel pathways rather than in a single genetic pathway. The observed effects could simply be additive rather than mechanistically linked.

R3_3

We thank the Reviewer for this comment and we agree with this possibility. Based on the findings from the experiments mentioned above, we now conclude that "*CYLD-1 is essential for normal lifespan and for lifespan extension mediated by disruptions in the insulin/IGF-1 signalling*". We had not supported a mechanistic link stemming from these experiments. We have further extended our analysis of the role of CYLD-1 to two distinct genetic paradigms of lifespan extension, these of *eat-2* and *glp-1* deficiency, representing calorie restriction and reproductive curtailment respectively. In both paradigms, depletion of *cyld-1* shortens lifespan (new Fig. 1F, G). These results provide additional evidence that CYLD-1 plays a generalized role in examples of lifespan extension ranging from disruption in the insulin/IGF-1 signalling to calorie restriction and reproductive curtailment.

4. The explanation of paralysis phenotypes (Figure 4C) is unclear. The authors conclude that paralysis is due to increased cholinergic transmission, but the alternative possibility of decreased GABAergic transmission is not addressed, even though compromised GABAergic synapse formation was observed in the mutants.

R3_4

We thank the Reviewer for the opportunity to further dissect our neurotransmission observations. To investigate whether GABAergic neurotransmission is perturbed under CYLD-1 deficiency, we utilized pentylenetetrazole (PTZ), an antagonist of the $GABA_A$ receptor. In the presence of PTZ, nematodes with underlying deficits in GABAergic neurotransmission display convulsions, reminiscent of an epileptic-like behaviour (10.21769/BioProtoc.2989). As positive controls, we used *unc-49(e407)* and *unc-43(tm1605)* mutant animals, harboring a mutation either in the nematode $GABA_A$ receptor UNC-49 or in the calcium/calmodulin-dependent protein kinase UNC-43, rendering the animals susceptible to PTZ-induced convulsions. Similar to wild type nematodes, *cyld-1* mutants did not display convulsions in the presence on PTZ (new Fig. S2D, E), demonstrating intact GABAergic neurotransmission under *cyld-1* deficiency. Therefore, we attribute the neurotransmission defects revealed by aldicarb and levamisole assays upon *cyld-1* deficiency to defective cholinergic signalling.

5. The colocalization data in Figure 4A are not convincing. The quantification lacks clarity, and it is unclear how colocalization was measured and analyzed.

R3_5

We thank the Reviewer for pointing at this gap in the method section. We have included a description on the intensity plot generation. We are not sure why the Reviewer finds that the colocalization data are not convincing and if the quantification still lacks clarity. We have utilized LSM900 Airyscan 2 super-resolution microscopy to acquire the images, processed them using the ZEN Blue software and visualize the localization of CYLD-1 in synapses using the intensity profile plot of a single pixel line in FIJI.

6. The criteria for defining neurodegeneration (Figure 3) are not well explained. There is no clear description of how neurodegeneration was quantified, and no supporting references are provided to justify the criteria used.

R3_6

We thank the Reviewer for this comment. We have now included analysis of the neuronal defects during ageing using the independent (outcrossed) *cyld-1(ok3637)* mutant strain as well (please refer also to Reviewer 1, comment 4, R1_4), showing similar degree of neurodegeneration to *cyld-1(tm3768)* animals. Our degeneration analysis focuses on the collective assessment of morphological aberrations associated with ageing, including fragmentation and defasciculation of ventral and dorsal nerve cord, missing or fragmented commissures and blebbing of neuronal processes (10.1073pnas.1533448100, 10.1523/JNEUROSCI.2911-10.2010, 10.1371/journal.pone.0031321). We now explicitly mention the above criteria in the method section including the respective references.

7. Allelic differences between cyld-1(tm3769) and cyld-1(ok3637) are not addressed. The reduced lifespan is more pronounced in the tm3769 allele, yet the differences between the two alleles are not explained. Additional data on the ok3637 allele should be included in Figure S1 for clarity.

R3_7

We thank the Reviewer for pointing this out, in line with Reviewer 1, comment 1 (please refer to comment R1_1.1 as well). The exact ok3637 variation (strain RB2610) was not annotated before. Briefly, we have mapped and report a deletion of 858bp, spanning the area chrIII:9911587-9912444 (new Fig. 1A). Following its characterization, we outcrossed the RB2610 strain twice with wild type N2 resulting in the strain IR3371. The outcrossed strain shows identical lifespan reduction to the one with the *cyld-1(tm3768)* variation (new Fig. 1B).

March 25, 2026

Ref: NCOMMS-25-03716A

Dear Reviewers,

We would like to thank you for the positive feedback in our revised manuscript. We have additionally addressed the remaining points raised by the Reviewer 3. All changes made in this round of revision, including corrections of minor errors identified during the preparation of the Source data file, are either highlighted in the manuscript or detailed in the source data file. Overall, your valuable input guided our efforts to a substantially more solid study. A point-by-point response to each comment follows below (shown in **blue**).

We will be standing by for any further clarifications or information, pertinent to our final version that may be required. Thank you very much again, for your consideration of our manuscript.

Sincerely,

Nektarios Tavernarakis,

for the authors

Reviewer #1:

In this revised manuscript, Sotiriou and colleagues have now convincingly established a role for CYLD-1 catalytic activity in aging. The authors have made concerted efforts to bolster their conclusions with substantial and well-controlled experiments. As examples, they have clarified the necessity rather than sufficiency of cyld-1 in aging. They have provided two additional paradigms of aging analyses. The authors have further clarified their findings in both autophagic flux and neuronal expression of CYLD-1. Finally, the authors have supported with outcrossed alleles the neuroprotective role of CYLD-1 in aging. I congratulate the authors on a clear study. They have addressed all of my critiques and I have no further objection.

We sincerely thank the reviewer for the positive feedback on our revised manuscript. We are particularly grateful that the reviewer's comments on our observations in ageing and autophagic-lysosome system led to an expanded and more solid study.

Reviewer #2:

The authors responded to all of my concerns I raised for the original manuscript in a satisfactory manner and the revised manuscript is acceptable for publication.

We sincerely thank the reviewer for shaping a more concrete and robust study. We would like to specifically mention the substantial reviewer's contribution on dissecting the role of autophagy in neurotransmission and cognitive functions under *cyld-1* deficiency as well as the CYLD's cell autonomous function in neurons and hypodermis.

Reviewer #3:

The manuscript by Sotiriou et al. has been substantially improved in the revised version, particularly with respect to clarifying the genetic pathways involving cyld-1 and strengthening the analyses related to autolysosomal/lysosomal function. However, several concerns still need to be addressed before the manuscript can be accepted.

We sincerely thank the reviewer for the positive evaluation of our revised manuscript. We highly appreciate the reviewer's contribution in aiding to further delineate the role of *cyld-1* in ageing and the meticulous revision rectifying both experimentation and result interpretation. Please find below our comments addressing the concerns raised in the second round of revision.

1.Have the authors tested whether systemic or neuron-specific overexpression of cyld-1 is sufficient to extend lifespan and/or preserve learning and memory during aging? Such gain-of-function experiments would help establish whether cyld-1 is not only necessary but also sufficient for promoting longevity and cognitive resilience.

We thank the reviewer for emphasizing this point. Yes, in our manuscript we had included lifespan data using *cyld-1* overexpressing animals (*cyld-1p::mcherry::cyld-1*). The extrachromosomal arrays of such animals are typically formed by multiple copies of plasmids leading to overexpression. These animals do not show differences compared to WT animals, suggesting that overexpression of *cyld-1* is not sufficient to extend lifespan (Fig. 1C). We provide here survival data of additional independent transgenic lines that show similar results (Fig. R3_1). We incorporated this observation in the text of our revised manuscript.

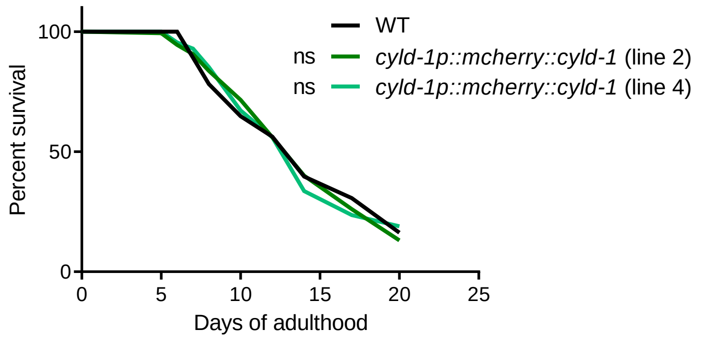

Fig. R3_1: These survival curves from additional independent lines of cyld-1p::mcherry::cyld-1 animals reveal that overexpression of (mcherry::)cyld-1 is not sufficient to extend lifespan (related to Fig. 1C).

In addition, as requested by reviewer 1 (point R1_1.3) and reviewer 2 (point R2_2) in the first round of revision, we have generated transgenic animals expressing *mcherry::cyld-1* under the control of the neuronal-specific promoter *rab-3* to rescue CYLD-1 activity only in neurons. However, neuron-specific *cyld-1* overexpression resulted in adverse effects such as developmental delay, internal hatching and compromised locomotion in the *cyld-1* mutant genetic background. This perturbed physiology is unfortunately present in *cyld-1*-overexpressing animals in wild type genetic background as well. We speculated that the developmental pattern of expression, the strength of the promoter (Fig. R1_1.3 of the first revision round and copied below, please see the aggregation pattern at the lower panel) or the ectopic expression in additional neurons elicited these deficits.

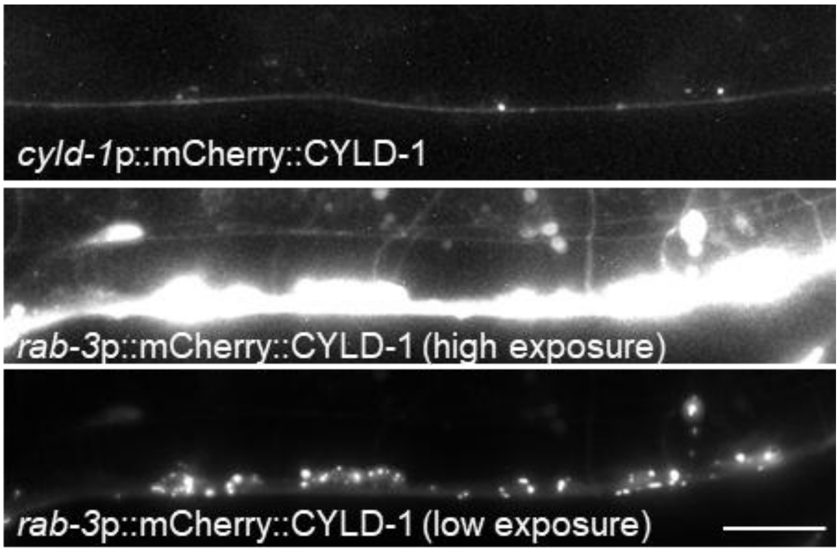

Fig. R1_1.3: Transgenic animals expressing mCherry::CYLD-1 under the control of the endogenous (upper panel) or the pan-neuronal promoter rab-3 (middle panel at high exposure and lower panel at low exposure). Images in the upper and middle panel were obtained using the same exposure settings, illustrating a stronger expression or retention of mCherry(::CYLD-1) under the control of the rab-3 promoter. Scale bar, 20µm.

2.Figure 1E–G: Revise the interpretation of these data. The results indicate that cyld-1 is not required for the lifespan extension conferred by daf-2, eat-2, or glp-1, and conversely that these longevity interventions do not substantially modify the shortened lifespan phenotype of cyld-1 loss-of-function animals. This apparent lack of epistasis is more consistent with cyld-1 acting in a parallel or largely independent pathway from IIS, dietary restriction, and germline signaling.

In addition, for Figure 1G, it is unclear whether there is a statistically significant difference between cyld-1(RNAi) and glp-1; cyld-1(RNAi). If there is no significant difference, this would instead support the interpretation that cyld-1 functions in the same pathway as glp-1, with glp-1 being epistatic to cyld-1. Please provide the relevant statistical comparisons (including exact p values) and clarify the conclusion accordingly.

We thank the reviewer for this observation. Indeed, CYLD-1 seems to act in parallel or largely independent pathways from *daf-2*, *eat-2* and *glp-1* interventions. We provided the respective statistical comparisons in all paradigms of Fig. 1 (D-G) and Supplementary Table 3, and clarified the above point in the revised manuscript accordingly.

3.Figure 4F–H: The interpretation of these data needs to be revised. While the unc-47 promoter–driven SNB-1 puncta density and fluorescence intensity appear decreased in cyld-1 mutants, the authors conclude that "these observations suggest that cyld-1–deficient animals preserve intact GABAergic neurotransmission despite their phenotypic alterations in GABAergic synapses (Fig. 4F–H)." This statement seems overstated given the observed reductions. Please clarify whether these changes are statistically significant, and if so, discuss the possibility that GABAergic presynaptic function may be partially impaired, rather than fully intact. If the differences are not significant, the authors should explicitly state this and temper the conclusion accordingly.

We thank the reviewer for this addition. This statement derives from the PTZ assay showing intact GABAergic neurotransmission under CYLD-1 deficiency. Indeed, the differences in puncta number and

intensity of GABAergic synapses were statistically significant (Fig. 4F-H). Therefore, we clarified that possible GABAergic functional defects correlated with the phenotypic alterations under *cyld-1* deficiency may be present, although the presynaptic GABAergic neurotransmission remains intact as evaluated by the $GABA_A$ receptor competition (PTZ) assay.

4.Figure 5I: The reported statistics appear inconsistent with the plotted data. The confidence intervals for lgg-2 and cyld-1; lgg-2 appear non-overlapping, yet the authors indicate that the comparison is not significant. Please clarify the statistical test used (including exact p values and any multiple-comparison correction, if applicable), and ensure that the confidence intervals and significance annotations are presented consistently.

We thank the reviewer for this observation. The data have been analysed with one-way ANOVA with Tukey's multiple comparison test and error bars indicate SEM, as stated in the figure legend and similar to the rest of the respective graphs in the manuscript. Please find below the graph with numerical p values, showing the confidence intervals which overlap (Fig. R3_4).

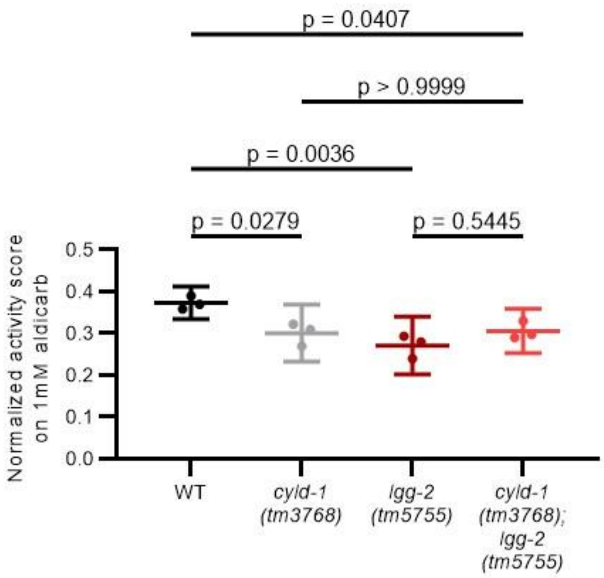

Fig. R3_4: This graph corresponds to Fig. 5I graph using confidence intervals instead of SEM. Please note that the different range of y-axis is used to include the confidence intervals ranges that differ from the ones of SEM for the same values of activity.

Finally, please note that during the preparation of the Source data file, we identified a numerical error in one experimental repeat of *lgg-2(tm5755)* condition and have updated the graph accordingly in the revised manuscript. This correction does not affect the interpretation and conclusions of the experiment.

In summary, additional statistics were applied in Fig. 1D-G, corrected data points with unaffected statistics were applied in Fig. 3D, 4K, 6C, S3G and corrected data points with minor changes in statistics were applied in 5I, 6J, S3H and S3I.
